# Transient Receptor Potential-Vanilloid (TRPV1-TRPV4) Channels in the Atlantic Salmon, *Salmo salar*. A Focus on the Pineal Gland and Melatonin Production

**DOI:** 10.3389/fphys.2021.784416

**Published:** 2022-01-07

**Authors:** Laura Gabriela Nisembaum, Guillaume Loentgen, Thibaut L’Honoré, Patrick Martin, Charles-Hubert Paulin, Michael Fuentès, Karine Escoubeyrou, María Jesús Delgado, Laurence Besseau, Jack Falcón

**Affiliations:** ^1^Sorbonne Université (SU), CNRS, Biologie Intégrative des Organismes Marins (BIOM), Banyuls-sur-Mer, France; ^2^Conservatoire National du Saumon Sauvage, Chanteuges, France; ^3^SU, CNRS Fédération 3724, Observatoire Océanologique, Banyuls-sur-Mer, France; ^4^Departamento de Genética, Fisiología y Microbiologia, Facultad de Biología, Universidad Complutense de Madrid, Madrid, Spain

**Keywords:** Atlantic salmon, temperature, pineal organ, melatonin, transient receptor potential vanilloid (TRPV), TRPV1, TRPV4

## Abstract

Fish are ectotherm, which rely on the external temperature to regulate their internal body temperature, although some may perform partial endothermy. Together with photoperiod, temperature oscillations, contribute to synchronizing the daily and seasonal variations of fish metabolism, physiology and behavior. Recent studies are shedding light on the mechanisms of temperature sensing and behavioral thermoregulation in fish. In particular, the role of some members of the transient receptor potential channels (TRP) is being gradually unraveled. The present study in the migratory Atlantic salmon, *Salmo salar*, aims at identifying the tissue distribution and abundance in mRNA corresponding to the TRP of the vanilloid subfamilies, TRPV1 and TRPV4, and at characterizing their putative role in the control of the temperature-dependent modulation of melatonin production—the time-keeping hormone—by the pineal gland. In *Salmo salar*, TRPV1 and TRPV4 mRNA tissue distribution appeared ubiquitous; mRNA abundance varied as a function of the month investigated. *In situ* hybridization and immunohistochemistry indicated specific labeling located in the photoreceptor cells of the pineal gland and the retina. Additionally, TRPV analogs modulated the production of melatonin by isolated pineal glands in culture. The TRPV1 agonist induced an inhibitory response at high concentrations, while evoking a bell-shaped response (stimulatory at low, and inhibitory at high, concentrations) when added with an antagonist. The TRPV4 agonist was stimulatory at the highest concentration used. Altogether, the present results agree with the known widespread distribution and role of TRPV1 and TRPV4 channels, and with published data on trout (*Oncorhynchus mykiss*), leading to suggest these channels mediate the effects of temperature on *S. salar* pineal melatonin production. We discuss their involvement in controlling the timing of daily and seasonal events in this migratory species, in the context of an increasing warming of water temperatures.

## Introduction

In ectotherms, metabolism, physiology and behavior rely on the external temperature (Angilletta et al., 2002; Storey and Tanino, 2012; Salin et al., 2016). In teleost fish, temperature can straightly affect the energetic metabolism, the endocrine regulations and related functions (e.g., food intake, stress responses, immunity), locomotor activity, sex determination and more (Quigley and Hinch, 2006; Ospina-Àlvarez and Piferrer, 2008; Steinhausen et al., 2008; Ibarz et al., 2010; Little et al., 2013; Chadwick et al., 2015). Together with photoperiod (the alternation of light and darkness of the 24 h cycle), temperature oscillates on a daily and annual basis. Both, photoperiod and thermoperiod provide rhythmic information, which is captured and transduced into internal time-keeping rhythmic messages. These messages allow synchronizing the fish metabolic, physiological, and behavioral rhythms to the daily and annual variations of the environment, including locomotor activity, sleep/rest, feeding, vertical migration, eggs production and laying for the former, growth, reproduction, and horizontal migration for the latter (Pankhurst and Munday, 2011; Villamizar et al., 2012; Falcón and Zohar, 2018).

The pineal organ of fish plays a central role in time decoding. Its epithelium possesses cone-like photoreceptor cells that transduce the photoperiodic and thermoperiodic information (Falcón, 1999; Falcón et al., 2007). In response to the alternation of light (L) and darkness (D) of the 24 h LD cycle, the pineal photoreceptor cells produce two messengers: (i) an excitatory neurotransmitter (glutamate) released at the synaptic junctions established with neurons that project in different brain centers (Meissl et al., 1986); (ii) a neurohormone—melatonin—released into the blood stream and cerebrospinal fluid (CSF) (Falcón, 1999; Falcón et al., 2007). Both messengers are produced in higher amounts at night than during day, and both are also modulated by the external temperature (Zachmann et al., 1992; Tabata and Meissl, 1993; Tabata et al., 1993; Thibault et al., 1993).

Melatonin synthesis from serotonin involves two enzymatic steps catalyzed, successively, by the arylalkylamine *N*-acetyltransferase [AANAT (EC 2.3.1.87): serotonin → *N*-acetylserotonin] and the acetyl serotonin-*O*-methyltransferase [ASMT (EC 2.1.1.4): *N*-acetylserotonin → melatonin] (Falcón, 1999; Falcón et al., 2007). Fish may express two or three AANAT isoforms: AANAT1a and/or AANAT1b, expressed in the retina and other central and peripheral areas, and AANAT2, which is pineal specific (Cazaméa-Catalan et al., 2014; Paulin et al., 2015). In the majority the species investigated, an intra-photoreceptor circadian molecular clock entrains *aanat2* gene expression (Falcón et al., 2007). Photoperiod acts at two levels allowing, on the one hand, synchronizing the clock and, on the other hand, controlling AANAT2 protein levels and enzymatic activity, so that duration of the melatonin production increase fits the duration of the night (Falcón et al., 2007). Salmonid species are an exception as no functional circadian clock has been identified so far in their pineal organ. The mechanisms underlying the photoperiodic regulation of pineal AANAT2 activity and melatonin production are quite well understood. The transduction of light information at the apex of the photoreceptor cell induces an intensity-dependent cell hyperpolarization. Conversely, the cell is depolarized in the dark, allowing the opening of cell-membrane voltage-gated Ca^2+^ channels (VGCC). The consequent increase in [Ca^2+^]_i_ at night activates the production of cyclic AMP (cAMP) and both, [Ca^2+^]_i_ and cAMP, contribute to stimulate AANAT2 protein synthesis and accumulation, with a subsequent increase in AANAT2 enzyme activity (Falcón et al., 2007). Upon illumination the whole process is reversed and AANAT2 protein is degraded.

How temperature information modulates the amplitude of the nocturnal production of melatonin by the pineal gland is far less understood. Temperature probably acts both at a molecular and a cellular level. (1) *At the molecular level*: the AANAT2 enzyme is a target because recombinant AANAT2 enzyme activity responds *in vitro* to temperature changes (Cazaméa-Catalan et al., 2012, 2013). And, specific amino-acid sequences within the AANAT2 protein sequence determine both, protein stability and enzyme catalytic efficiency. This AANAT2 protein response is species-specific and related to the temperature habitat of the fish. This is not the case of recombinant AANAT1a and AANAT1b, which activities increase linearly with temperature from 0 to 37°C and then drop to zero value at higher temperatures, whatever the species studied. (2) *At the cellular level:* there are arguments to believe that temperature effects are mediated through the Ca^2+^/cAMP regulatory pathway mentioned above. Indeed, in organ culture, cAMP content and AANAT2 activity display superimposed bell-shaped responses to changing temperatures of incubation in the trout *(Oncorhynchus mykiss*) and the pike (*Esox lucius*) (Thibault et al., 1993; Falcón et al., 1996; Benyassi et al., 2000); the responses are species-specific and match approximately the fish thermal preferences. In addition, one study in the trout indicated that the thermo-sensitive transient receptor potential (TRP) channels from the vanilloid family (TRPV) are involved. These channels modulate the entry of Ca^2+^ within cells. Indeed, TRPV1 and TRPV4 channels are expressed exclusively in the photoreceptor cells of the pineal gland (Nisembaum et al., 2015). Moreover, the study showed that the *in vitro* secretion of melatonin by isolated trout pineal glands in culture was modulated by TRPV1 and TRPV4 agonists and antagonists in a temperature-dependent manner: while TRPV1 mediated responses at intermediate temperatures (∼16°C), it was suggested that TRPV4 operated at colder temperatures (∼8°C). Altogether, it was concluded that light and temperature both interact to modulate [Ca^2+^]_i_ and consequently the cAMP-dependent control of melatonin secretion by the pineal photoreceptor cells (Nisembaum et al., 2015).

The present study was undertaken to confirm and extend the information gained from the study in the trout to another salmonid, the Atlantic salmon *Salmo salar*, as a part of a project aiming at better understanding the impact of the global temperature rise on this threatened population of the Loire/Allier basin (France). Here we provide the first information on (i) the tissue distribution of TRPV1 and TRPV4 mRNA in salmon smolts, and variations in their relative abundance at two different months of their first year, (ii) their cellular localization in the pineal gland and retina of adult fish using *in situ* hybridization (ISH) and immunohistochemistry (IHC), and (iii) the impact of TRPV1 and TRPV4 agonists and antagonists on melatonin secretion by isolated pineal glands *in vitro*.

## Materials and Methods

### Animals

**FIGURE 1:**
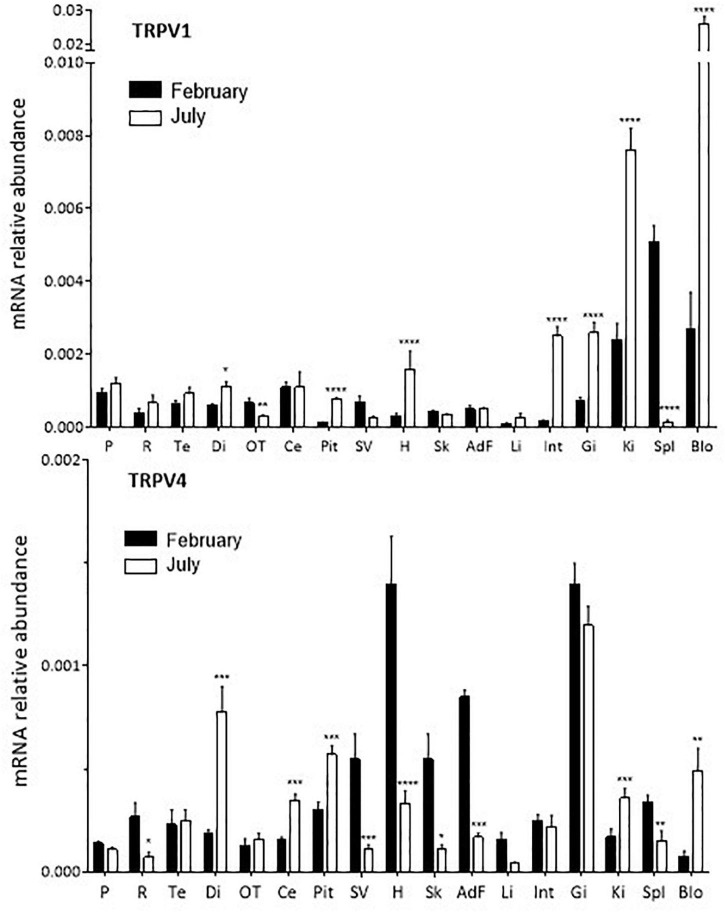
Relative quantitative variations of TRPV1 and TRPV4 expression in *S. salar* smolts in February and July. For each tissue the values are normalized to those obtained with the reference gene *ef1*α as indicated in “Materials and Methods” section. Mean ± SEM, *n* = 4–8. The Student’s *t* test compares the February vs. the July values: *P < 0.05, **P < 0.01, ***P < 0.0001, ****P < 0.00001. *AdF, adipose fin; Blo, blood; Ce, cerebellum; Di, diencephalon; Gi, gills; H, heart; Int, intestine; Ki, kidney; OT, optic tectum; P, pineal organ; Pit, pituitary; R, retina; Sk, skin (including the lateral line); Spl, spleen; SV, saccus vasculosus.*

Atlantic salmon (*Salmo salar* L.) were raised indoors at the *Conservatoire National du Saumon Sauvage* (CNSS, France, 45°N^1^). The CNSS hatchery produces juvenile salmon, which are to be released at different developmental stages in areas of the Loire-Allier basin requiring supplementation, as part of a restoration program to enhance the native Atlantic salmon population. The fish used for the mRNA detection in the different tissues were from a group of smolts used in a previous study (Nisembaum et al., 2020). Briefly, they were reared indoors under simulated natural conditions of photoperiod and natural temperature [February: 11L_(07:30–18:30)_/13D and 4°C; July: 16L_(06:00–22:00)_/8D and 15°C]; they weighed ∼30 g in February, and ∼80 g in July. Details are provided in Figures 1, 2 of Nisembaum et al. (2020). The fish used for the ISH and IHC (250 g *b.w*.), and *in vitro* pharmacological studies (600 g *b.w*.) were from the hatchery’s broodstocks (each year, a batch of ∼8 months old fish exceeding 145 mm is retained in the hatchery for 3 years to become the future broodstocks). The 1st generation hatchery-reared progeny was obtained from wild male and female adult salmon caught in the Allier River (620 km from the Loire estuary). Rearing occurred under simulated natural photoperiod and natural temperature, and standard hatchery conditions as described elsewhere (Martin et al., 2012; Nisembaum et al., 2020). At their juvenile stage fish were distributed in four 9 m^3^ cylindrical tanks (depth 0.5–0.7 m) at a density that did not exceed 10 kg/m^3^. The tanks were supplied with running water from the Allier River, at a flow of 3 l/s until April, which was progressively increased to 7 l/s when water temperature reached values above 13°C. This ensured a concentration of dissolved oxygen higher than 7 mg/l (Martin et al., 2012). As detailed elsewhere (Nisembaum et al., 2020), all fish were fed during daytime using automatic feeders. The acclimation conditions at the CNSS were in accordance with the “Agreement N° B43 056 005” (Arrêté N° DDCSPP/CS/2016/40), and the experimentation followed the guidelines and regulations approved by the “Ethics Committee for Animal Experiment of Languedoc-Roussillon (C2EA-LR/C2EA-36)” N° A6601601, and the European Union regulations (European directive 2010/63/EU).

**FIGURE 2:**
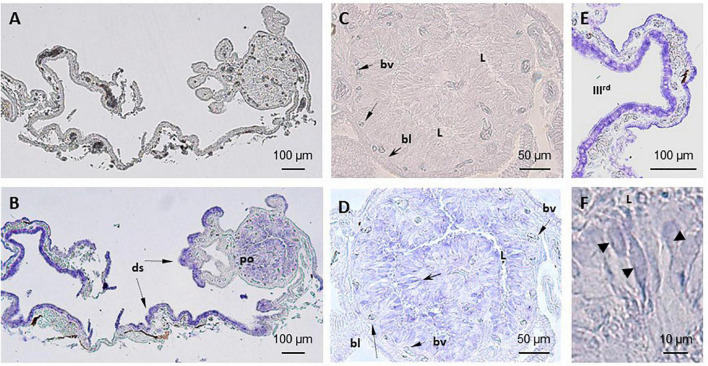
Localization of TRPV1 mRNA in *S. salar* pineal complex by *in situ* hybridization (ISH). **(A–C)** No labeling is seen in sections treated with the sense probe. **(B,D–F)** Anti-sense probes. **(B)** Shows the entire pineal complex and the presence of transcripts in the pineal organ (po) and dorsal sac (ds). Higher magnifications show the signals are in the photoreceptors (black arrow, **(D)** and in the cells in the border of the third ventricle in the dorsal sac **(E)**. **(D)** Shows a section through the pineal vesicle and labeled cells located around the lumen (L), which appear distant from the blood vessels (bv) and basal lamina (bl). This location corresponds to that occupied by the photoreceptor cells also identified by their segmented shape as seen in **(F)** (arrow heads). **(E)** Shows cells of the dorsal sac tissue that surrounds the third ventricle (IIIrd).

### Sampling

Sampling was performed in the morning (between 10:00 and 12:00). Animals were anesthetized with 2-phenoxy−ethanol (0.5 mg/l) and then killed by decapitation. Tissues and organs were sampled and immediately dipped in the appropriate solution: (i) RNA later, first at + 4°C for 24 h, and then at −80°C until RNA extraction, for the real time quantitative PCR (RT-qPCR) studies; (ii) ice cold fixative [freshly prepared 4% paraformaldehyde (PFA) in phosphate buffer (PB; 0.1 M, pH 7.4)] for the ISH and IHC studies; (iii) ice cold freshly prepared culture medium for the culture of pineal organs and pharmacology studies.

### Reverse Transcription Quantitative Real Time PCR

Total RNA extraction from adipose fin, blood, cerebellum, diencephalon, gills, heart, intestine, kidney, optic tectum, pineal organ, pituitary, retina, skin (including the lateral line), spleen, and *saccus vasculosus*, was performed using an automated system and kit (Maxwell^®^; Promega, Charbonnières-les-Bains, France) according to the manufacturer’s protocol. Retro-transcription was performed with 1 μg of RNA for all tissues except for the *saccus vasculosus* (for which 0.5 μg were used), using the PrimeScript™ 1st strand cDNA synthesis kit (Takara Bio Inc., Ozyme, Saint-Quentin-en-Yvelines, France). The abundance of the mRNA corresponding to the genes studied [*trpv1* (NM_001140498.1; Leong et al., 2010), *trpv4*, (KJ135123.1; Nisembaum et al., 2015) and the reference gene *ef1*α (NM_001141909.1; Leong et al., 2010)] was quantified using a Light-Cycler™ system 2.0 (Roche; Meylan, France). The reactions were performed in a 20 μl final volume, containing 10 μl of LightCycler-FastStart DNA Master SYBR-Green I™ Mix (Roche Diagnostics; Meylan, France), 0.2 μM specific primers (Eurofins, Ebersberg, Germany; Table 1), and 2 μl of 1/5 diluted cDNA. The amino-acid sequences amplified are detailed in Supplementary Figure 1. The amplification protocol was as follows: 1 cycle of enzyme activation at 95°C for 3 min, and 40 cycles consisting in 95°C for 3 s, 60°C for 30 s and 72°C for 20 s. All samples were analyzed in duplicates and the relative expression (ΔΔCT) was performed according to Livak and Schmittgen (2001), taking into account the efficiency of the PCR reactions (Pfaffl, 2001). The efficiency of the amplification for all the genes investigated was around 100%. The specificity of the amplification reactions was confirmed by the melting temperature in each sample, through a melting curve protocol at the end of the 40 cycles of amplification, and by the size of the PCR products, obtained in an agarose gel. The data are presented as the mean ± SEM of *n* = 4–8 samples.

**TABLE 1:** Primers used for the RT-qPCR and for the preparation of the ISH probes.

RT-qPCR	Accession number		Primer sequences 5′→3′	Product (bp)
*ef1*α	NM_001141909.1	F	CCTACAGCCAGAAGCGTTTT	169
		R	TCGACCTTCCATCCCTTGAA	
*trpv1*	NM_001140498.1	F	CGTCCTGCTGAAGGCTCTA	122
		R	TGTCTGTGTATGCAGCATTTACAA	
*trpv4*	KJ135123.1	F	GAGAATCGCCATGAGATGC	155
		R	TCGGATGGGTGGTAGTA	
ISH probes				
*trpv1*	NM_001140498.1	F	AGCATCTGGAAACTACAGCG	696
		R	TGCTCAACACAGATTGCAGT	
*trpv4*	KJ135123.1	F	GGTGAGCTGCCTCTGTCG	302
		R	ACCCCAATTTTTCCCAGTTTGG	

*bp, base pairs; F, forward; R, reverse.*

### *In situ* Hybridization and Immunohistochemistry

Pineal glands from adult fish (250 ± 50 g *b.w*.), were used for these assays. After 24 h fixation in PFA (see above) the samples were washed in PB, dehydrated in graded ethanol series (70, 95, 100%), dipped 3 min in toluene and then in Paraplast^®^ (at 60°C); after 15 h of impregnation, they were embedded in a new bath of Paraplast^®^. Eight micrometers thick sections (using a MicroM HM 340^E^ microtome) were layered on glass slides (coated with a 2% solution of 3−aminopropyl-triethoxy-silane). The ISH and IHC (3 glands for each procedure) were performed on sections that were successively deparaffinized in toluene, rehydrated (through descending ethanol series) and placed in PB saline (PBS).

The ISH was performed on proteinase K treated sections (5 mg/ml; for 6 min at 37°C), using digoxigenin (DIG) labeled probes. The preparation of the riboprobes and the ISH procedure were as detailed elsewhere using a commercial kit (Roche-Diagnostics DIG labeling kit) (Besseau et al., 2006; Nisembaum et al., 2015; Paulin et al., 2015). The primers sequences for the preparation of the probes are given in Table 1 and Supplementary Figure 1.

The IHC was performed on pineal organs and retinas. Pineal and retinal sections were dipped in a 3% H_2_O_2_ solution (in PBS) for 10 min in the dark and rinsed in PBS. The first antibody, from a commercial origin (Abcam, Cambridge), was a polyclonal rabbit anti-zebrafish antibody directed against either TRPV1 (1/100), or TRPV4 (1/500); it was applied for 16 h at + 4°C. Revelation was then performed using a commercially available kit (IHC Select^®^ HRP/DAB) that contained the second antibody (anti-rabbit coupled to horseradish peroxidase [HRP]) and the substrates [diaminonobenzidine (DAB) and H_2_O_2_], and following the protocol instructions. The first antibody was omitted in the control sections as indicated in the kit instructions.

### *In vitro* Culture of Pineal Glands and Pharmacological Assays

Pineal glands of adult fish (600 ± 50 g *b.w.*) were cultured in 24-wells culture plates (Nunclon™ Surface; VWR International, Fontenay-sous-Bois, France) as detailed elsewhere (Bégay et al., 1992; Nisembaum et al., 2015). Each well contained one gland in 500 μL of medium (RPMI 1640 without phenol red) complemented with penicillin (100 U/ml), streptomycin (100 μg/ml), glutamine (2 mM), and fungizone (2.5 μg/ml). The culture plates were placed in a MIR-154 incubator (Sanyo; Osaka, Japan) under the photoperiod and temperature conditions the fish had been acclimated to. The media were renewed every 24 h. After 48 h, the pineal glands (*n* = 7–8/group) were placed (at 12:00) for 6 h in the dark, in the presence of vehicle or increasing concentrations of either capsaicin (TRPV1 agonists) or 4α-Phorbol 12,13-didecanoate (4αPDD; TRPV4 agonist); these experiments were performed in the absence or presence of 1 μM of the respective antagonists (1 μM capsazepine for TRPV1, 10 μM ruthenium red for TRPV4). More details are given in the results section and legend of the figures. Ten millimolar stock solutions were prepared in the appropriate vehicle [100% methanol (capsazepine), DMSO (4αPDD), ethanol (capsaicin) or ultrapure water (ruthenium red)]. The final solvent concentration did not exceed 0.2% and controls contained an equivalent amount of vehicle. At the end of the 6 h, the culture media were collected and frozen at −20°C until melatonin quantification. All experiments were run in duplicate, the data are presented as the mean ± SEM.

### Melatonin Quantification

The concentration of melatonin released in the culture medium was determined by High Performance Liquid Chromatography (HPLC) using either (1) a 100 × 4.6 mm C8 reversed-phase analytic column (Waters Spherisorb; Milford, MA) with particles size of 3 μm and an Agilent fluorescence detector (1,100 series; Santa Clara, CA, United States) or (2) a 125 × 4.6 mm C18(2) reversed phase analytic column (Luna, Phenomenex; Le Pecq, France), with particles size of 5 μm and a Dionex™ ULTIMATE™ 3100 fluorescence detector (Thermo Scientifique™; Villebon-sur-Yvette, France). Two to ten microliter of each sample were directly injected in the HPLC system. The excitation and emission wavelengths were 280 and 340 nm, respectively; the column temperature was maintained at 30°C; the mobile phase consisted of 0.1 M Na_2_HPO_4_ containing 10% (protocol 1) or 20% (protocol 2) acetonitrile; pH was adjusted to 6.5 with orthophosphoric acid. The mobile phase flow was 1 ml/min (protocol 1) or 1.5 ml/min (protocol 2), and the retention times of melatonin standards and samples were of ∼31 min (protocol 1) and ∼7 min (protocol 2). Standard curves were prepared after diluting a stock melatonin solution (10 mM; prepared in 100% methanol) in HPLC- grade water.

### Statistics and Graphics

The analysis included one- or two-way ANOVA followed by the Holm-Sidak or Sidak *post hoc* tests depending on the dataset. Individual means were compared using the Two-tailed Student’s *t*-test. Drawings and statistics were performed using the Prism.v6 (GraphPad™ Software Inc., San Diego, CA).

### Compounds and Chemicals

3−aminopropyltriethoxysilane, 4-α-Phorbol 12,13-didecanoate (4αPDD), capsaicin, capsazepine, EDTA, eugenol, fungizone (Amphotericin B), L-glutamine-penicillin, paraplast^®^, 3−aminopropyltriethoxysilane, streptomycin solution, ruthenium red, and RPMI culture medium were from Sigma-Aldrich (Saint-Quentin Fallavier, France). Melatonin standard was from Acros Organics™ (Fisher Scientifics, Villebon- sur-Yvette, France). Acetonitrile and hydrogen peroxide solution-HPLC grade were from Fisher Scientifics (Villebon- sur-Yvette, France). RNA later was from Life technologies SAS (Saint Aubin, France). The DIG labeling kit was from Roche Diagnostics (Meylan, France). The polyclonal rabbit anti-zebrafish antibodies (anti-TRPV1, Ab68969; anti-TRPV4, Ab69094) were from Abcam (Cambridge, England). The IHC revelation kit (IHC Select^®^ HRP/DAB) was from Merck-Millipore (Molsheim, Alsace, France).

## Results

### Sequences Analyses

The amino acid sequences corresponding to *S. salar* TRPV1 and TRPV4, as well as their alignment with the corresponding TRPV sequences from other vertebrates, are given in Supplementary Figures 1–3. The sequences amplified by the primers used in this study (Table 1) are also highlighted (Supplementary Figure 1). The couples of primers, chosen for either the qPCR or the ISH, displayed no significant alignment.

### Tissue Distribution of TRPV1 and TRPV4 Channels

TRPV1 and TRPV4 mRNA were ubiquitously distributed in *S. salar* brain and peripheral tissues (Figure 1 and Supplementary Figure 4). However, their relative abundance varied from one tissue to another (Supplementary Figure 4). TRPV1 mRNA abundance was particularly high in the kidney, spleen and blood cells at both months investigated, while TRPV4 mRNA was particularly abundant in the gills (February and July), adipose fin and heart (February only).

In some tissues, variations in abundance were found between the February and July samples (Figure 1). *TRPV1:* abundance was 2–10-fold higher in July compared to February in the diencephalon, pituitary, heart, intestine, kidney, gills, and blood cells. The opposite held true for the *optic tectum*, spleen and *saccus vasculosus*, while no variation was seen in the case of the pineal organ and the retina. *TRPV4*: significantly higher amounts were detected in February vs. July in the retina, *saccus vasculosus*, heart, skin (including lateral line), liver, spleen and adipose fin; conversely, amounts were higher in July in the diencephalon, cerebellum, pituitary, kidney and blood cells. Again, no change was detected in the pineal organ.

### TRPV1 and TRPV4 in the Pineal Gland

#### *In situ* Hybridization

**FIGURE 3:**
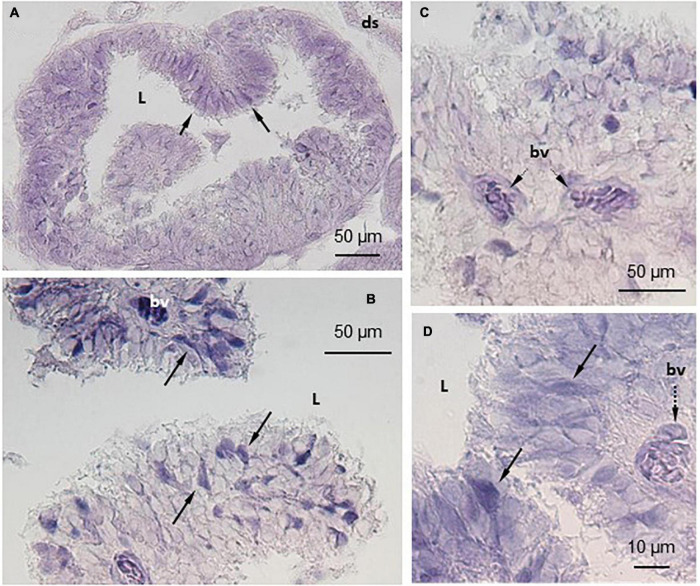
Localization of TRPV4 mRNA in *S. salar* pineal complex by *in situ* hybridization (ISH). The anti-sense probes allowed localization of TRPV4 mRNA in the pineal photoreceptor cells that are in contact with the pineal lumen (L) and displaying the typical segmented shape **(A,C)**. Some cells appeared more intensely labeled than others (arrows in **B,D**). The cells in the blood vessels (bv) were also intensely labeled **(C,D)**. ds, dorsal sac.

Similar observations were made with either the TRPV1 or the TRPV4 mRNA anti-sense probes (Figures 2, 3). A labeling was observed in the cell bodies of the pineal photoreceptors (TRPV1: Figures 2B,D,F; TRPV4: Figures 3A,B,D). The TRPV1 probe also labeled cells from the dorsal sac, the tissue adjacent to the pineal gland (Figures 2B,E), while the TRPV4 probe labeled cells in the blood vessels (Figures 3C–D). No signal was observed in the negative controls, hybridized with the sense probes (TRPV1: Figures 2A,C; TRPV4: not shown).

#### Immunohistochemistry

**FIGURE 4:**
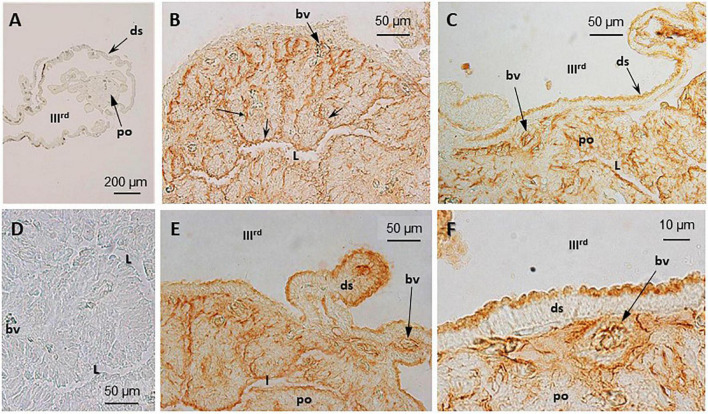
Immunohistochemical (IHC) detection and localization of TRPV1- and TRPV4-like compounds in the pineal complex of *S. salar*. Sections were treated as indicated in “Materials and Methods” section. No labeling is seen in the control sections **(A)**, TRPV1; **(D)**, TRPV4 when the primary anti-body is omitted. Both the anti-TRPV1 **(B,E)** and anti-TRPV4 **(C,F)** treated sections displayed a similar labeling pattern. In the pineal organ (po) the brown deposits are seen in the most apical and basal parts that border, respectively, the pineal lumen (L), and the basal lamina (bl) and blood vessels (bv). The dorsal sac (ds) cells are also labeled in their apical part, bordering the IIIrd ventricle.

TRPV1- and TRPV4-like proteins were identified in the pineal organ and the dorsal sac using the corresponding antiserum (Figure 4). A similar pattern was obtained, irrespective of the antibody used (TRPV1: Figures 4A–C; TRPV4: Figures 4D–F). The labeling appeared intense at both the apical (pineal lumen) and basal parts of the epithelium. The apical part bathes in the CSF of the IIIrd ventricle. The basal part is close to the basal lamina and the blood vessels. Some blood cells also appeared labeled (Figures 4C,F). More faint brown deposits were also observed delimiting cells within the pineal epithelium. The labeling of the dorsal sac was concentrated in the most apical part of the cells that bath into the CSF of the IIIrd ventricle (Figures 4C,E,F). No staining was seen in the controls (Figures 4A,D).

#### Melatonin Secretion

**FIGURE 5:**
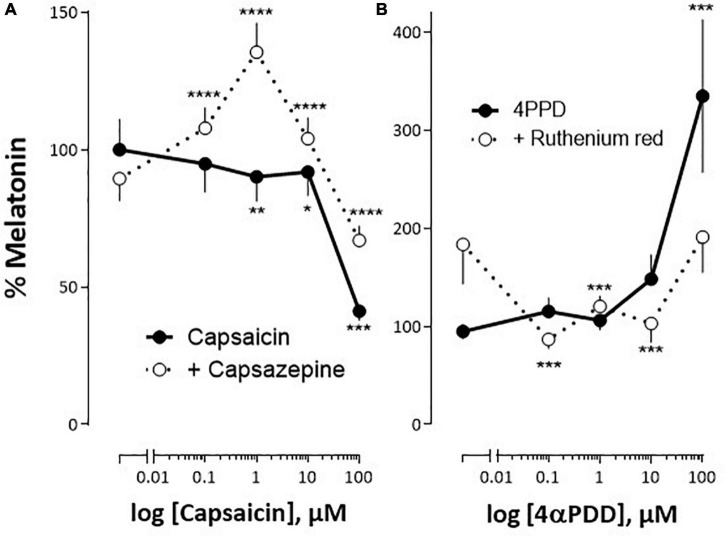
Impact of TRPV1 and TRPV4 analogs on *S. salar* pineal melatonin secretion *in vitro.* The pineal organs were cultured as indicated in “Materials and Methods” section. **(A)** The organs were challenged with increasing concentrations of capsaicin (TRPV1 agonists), in the absence (continuous line) or presence of 1 μM capsazepine (TRPV1 antagonist; interrupted line). **(B)** The organs were challenged with increasing concentrations of 4αPDD (TRPV4 agonist) in the absence (continuous line) or presence of 10 μM ruthenium red (TRPV4 antagonist; interrupted line). The 100% value corresponds to the melatonin concentration measured in the presence of vehicle only. Mean ± SEM of two independent experiments performed in April and October **(A)**, and May and October **(B)**, *n* = 16. The 2-way ANOVA indicated: **(A)** a significant effect of capsaicin (*P* < 0.00001), of the antagonist (*P* = 0.0015) and of their interaction (*P* = 0.023); **(B)** a significant effect of 4αPDD (*P* < 0.0001), no effect of the antagonist (*P* = 0.27) and an effect of their interaction (*P* = 0.013). *Post hoc* test compares means measured in the absence or presence of the antagonist: *P < 0.01, **P < 0.001, ***P < 0.0001, ****P < 0.00001.

In the dark, the release of melatonin by cultured pineal organs was modulated by different concentrations of the TRPV1 agonist, capsaicin: the very slight decrease observed in the presence of concentrations ranging from 0.01 to 10 μM capsaicin (not exceeding ∼10% at 10 μM), was followed by an abrupt (∼60%) decrease at the higher concentration (100 μM) (Figure 5A). This response to capsaicin was modified in the presence of the TRPV1 antagonist capsazepine (1 μM), which had no proper effect. In the presence of the antagonist, capsaicin became stimulatory at the low (0.1–1 μM), and inhibitory at the high (10–100 μM), concentrations used, resulting in a bell-shaped dose-response (Figure 5A).

The TRPV4 channel agonist, 4αPDD, induced a significant increase in melatonin secretion only at the highest concentrations used (100 μM; Figure 5B). The addition of 10 μM ruthenium red, an antagonist at the TRPV4 channel, counteracted this effect. It was noticeable that by itself ruthenium red tended to increase basal melatonin release in the dark, although this effect did not appear statistically significant.

### TRPV1 and TRPV4 in the Retina

**FIGURE 6:**
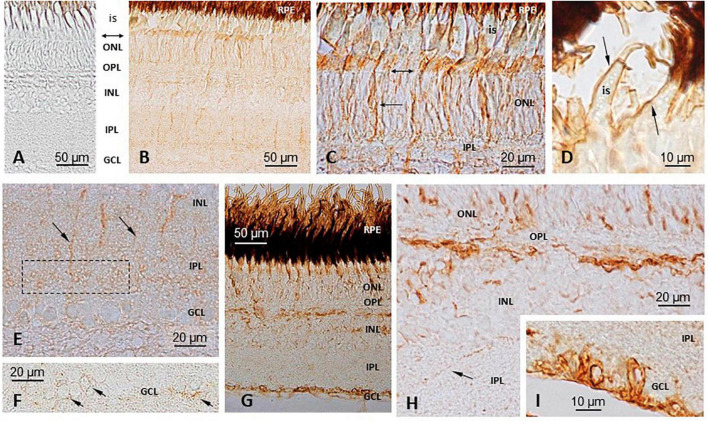
Immunohistochemical (IHC) localization of TRPV1 and TRPV4 in *S. salar* retina. **(A)** Negative control, with no primary anti-body **(D,G,H,I)** Primary anti-body against TRPV4. TRPV1 **(B,C,E,F)**. A weak immunoreactivity was observed at the levels of the outer nuclear layer (ONL), outer limiting membrane (double headed arrow in **C**), and inner segments (is) of the photoreceptors **(B,C)**. Some cells appeared more intensely labeled than others. A few vertical prolongations, probably corresponding to glial Müller cells, marked the whole height of the retinal epithelium from the basal part of the ONL down to the upper part of the ganglion cells layer (GCL) **(B,E)**. An area in the basal part of the inner plexiform layer (IPL) concentrated some labeling **(E)**. GCL cells also displayed some faint labeling in their periphery **(F)**. TRPV4 **(D,G–I)** A staining was seen in the ONL, with some cell bodies appearing more intensively marked than others **(G,H)**. At a high magnification the brown deposits were seen marking the membrane of the photoreceptors inner segments (is; **D**). A strong labeling was also seen in the upper part of the Inner nuclear layer (INL), possibly where the horizontal cells are **(G,H)**, while some scattered cells were also labeled deeper in the INL **(H)**. An intense staining was also observed in the cell bodies and axons of the GCL **(G,I)**. IPL, Inner plexiform layer; OPL, Outer plexiform layer; RPE, Retinal pigment epithelium.

Immuno-detected TRPV1 and TRPV4 proteins of the Atlantic salmon were found in all cell layers of the retina (Figure 6), while no staining was seen in the controls (Figure 6A).

#### TRPV1

The intensity of the labeling was weak. The immunoreactivity was observed at the levels of the outer nuclear layer (ONL), outer limiting membrane and inner segments of the photoreceptors (Figures 6B,C). Some cells appeared more intensely labeled than others. A few vertical prolongations of unknown origin marked the whole height of the retinal epithelium from the basal part of the ONL down to the upper part of the ganglion cells layer (GCL) (Figures 6B,E); this let us think they corresponded to Müller cells. An area in the basal part of the inner plexiform layer (IPL) concentrated some labeling (Figure 6E). In the GCL, some cells also exhibited some faint labeling at their periphery (Figure 6F).

#### TRPV4

The staining was seen in the ONL, with some cell bodies appearing more intensively marked than others (Figures 6G,H). At a high magnification, the brown deposits were seen marking the membrane of the photoreceptors’ inner segments (Figure 6D). A strong labeling was also seen in the upper part of the INL, possibly where the horizontal cells are located (Figures 6G,H), while some scattered cells were also labeled deeper in the INL (Figure 6H). Finally, an intense staining was observed in the cell bodies and axons of the GCL (Figures 6G,I).

## Discussion

The current investigation adds to the relatively few and scattered investigations on TRPV channels in fish, and extends to the Atlantic salmon previous investigations on the localization and role of TRPV1 and TRPV4 in the photosensitive pineal organ of the rainbow trout.

### TRPV1 and TRPV4 Exhibit a Widespread Distribution

Together with the previous studies [trout *O. mykiss* TRPV1 and TRPV4 (Nisembaum et al., 2015); chum salmon *Oncorhynchus keta* TRPV4 (Lee et al., 2021); tilapia *Oreochromis mossambicus* TRPV4 (Watanabe et al., 2012); half-smooth tongue sole *Cynoglossus semilaevis* TRPV4 (Shang et al., 2020); sea bass *Dicentrarchus labrax* (Bossus et al., 2011); zebrafish *Danio rerio* (Gau et al., 2013)], this study provides evidence that TRPV1 and TRPV4 are widely distributed in nervous and non-nervous tissues of fish. The experiments conducted here in *S. salar* at the months of February and July suggest variations in mRNA abundance may exist from a month to another in some tissues, as indicated by studies in the rainbow trout (Nisembaum et al., 2015). These differences might result from seasonal variations related to environmental changes (photoperiod and/or temperature) and/or developmental differences (fish studied here were developing yearlings) and/or smoltification (the fish were smolts in February and had achieved smoltification in July). More experimentation is needed to elucidate this point. These observations and the possible existence of differences among species or experimental procedures, make difficult any comparison concerning the levels of abundance. For example, a previous *in situ* hybridization study in *S. salar* allowed detection of TRPV1 and TRPV4 transcripts only in the telencephalon and optic lobes (Boltana et al., 2018), which contrasts with the present data showing expression in all brain areas, including the pineal gland, retina, pituitary gland, *saccus vasculosus*, telencephalon, diencephalon, optic tectum and cerebellum.

The variety of tissues expressing the TRPV channels is most probably related to the fact that these channels are multimodal effectors sensitive to a large number of stimuli including temperature (Patapoutian et al., 2003; Dhaka et al., 2006; Cohen and Moiseenkova-Bell, 2014; Li et al., 2020), ionic balance (Bossus et al., 2011; Seale et al., 2012), pressure and stretching (Liedtke and Kim, 2005; Watanabe et al., 2012; Startek et al., 2019), pH, ligands and ions [Ca^2+^, Mg^2+^] (Vriens et al., 2009), H_2_0_2_, lipids and lipid derived metabolites (arachidonic acid, anandamide, *N*-arachidonoyldopamine, lipoxygenase) (Leonelli et al., 2011; Zheng, 2013; Raboune et al., 2014; Cordero-Morales and Vasquez, 2018). It is beyond the scope of this study to discuss in depth the presence and role of TRPV1 and TRPV4 channels in the different tissues of *S. salar*. As detailed below, the main focus was *S. salar* pineal and, because the pineal gland and the retina are two homologous organs, derived from the same diencephalic origin (O’Brien and Klein, 1986), we also ran some parallel experiments in the retina.

### TRPV1 and TRPV4 in the Pineal Area

In the pineal epithelium both, TRPV1 and TRPV4 transcripts, were detected only in the photoreceptor cells. The photoreceptors were identified by (i) their position in the upper part of the pineal epithelium, bordering the lumen of the organ, in which their apical part protrudes (i.e., they are in direct contact with the CSF) and (ii) their shape and segmented organization. These results are comparable to those previously obtained in the pineal gland of *O. mykiss* (Nisembaum et al., 2015). In the Atlantic salmon, the IHC detection of the corresponding proteins suggested a localization in membranes rather than the cytosol, with the channels concentrating in the apical part of the photoreceptor cells, which bathes into the CSF. The position of the faint IHC labeling also seen within the epithelium suggests they might correspond to neuropil areas. These areas contain photoreceptor endings making synaptic contacts with the pineal second-order neurons. In this regard, it is interesting that an ISH study in the rat showed that TRPV1 was associated with the synaptic ribbons of the pinealocytes (Reuss et al., 2010).

*In vitro* studies have shown that in both, the rat and the trout pineal organs in culture, the production of melatonin was modulated by the TRPV1 agonist capsaicin, and the effects were antagonized by the TRPV1 antagonist capsazepine (Reuss et al., 2010; Nisembaum et al., 2015). Here we show that melatonin secretion is also modulated by TRPV1 analogs in the Atlantic salmon. The modulation of melatonin production by TRPV1 appears thus as an ancestral character. Of interest is the observation that capsaicin had similar dose-dependent bell-shaped effects in trout and salmon pineal glands; and, in both cases the optimal effect was obtained at the micromolar concentration of the agonist; but in *S. salar* the effects of the agonist were observed only in the presence of the antagonist. Another similarity between trout and salmon lies in their response to TRPV4 analogs. In both cases 4αPPD had no effect on melatonin secretion [except in the Atlantic salmon at the highest (0.1 mM) concentration used]; but, in the presence of the antagonist ruthenium red, 4αPPD induced a similar U-shaped dose-response curve in both rainbow trout and Atlantic salmon (statistically significant in the Atlantic salmon only). The complexity of the responses to the TRPV agonists and antagonists has been discussed in the rainbow trout study (Nisembaum et al., 2015). Altogether, we conclude that TRPV1 and TRPV4 contribute to modulate *in vitro* melatonin secretion in a similar manner in the rainbow trout and Atlantic salmon pineal organs.

The question raises to know what triggers TRPV opening and closure. We believe that temperature is one possible candidate because: (i) the *in vitro* pharmacological responses to the TRPV analogs in the trout and salmon pineal glands were quite similar, and it is known that at least capsaicin mimics the effect of elevated temperature in TRPV1 channel, (ii) in the trout the effects of the TRPV1 and TRPV4 antagonists depended on temperature (∼16°C for TRPV1 and ∼8°C for TRPV4) (Nisembaum et al., 2015), and (iii) the TRPV1 and TRPV4 sequences from both fish displayed high identity (97% for TRPV1 and 98% for TRPV4). This would agree with previous observation indicating that (i) the fish pineal gland and melatonin are involved in behavioral thermoregulation (Kavaliers and Ralph, 1980; Ekström and Meissl, 1997), as is also the case in lizards (other ectotherms; Ralph et al., 1979a,b; Skinner, 1991), and (ii) temperature modulates both the hormonal and nervous pineal outputs (Zachmann et al., 1992; Tabata and Meissl, 1993; Tabata et al., 1993; Thibault et al., 1993; Falcón et al., 2007). Altogether it is reasonable to believe that the TRPV1 and TRPV4 channels of the Atlantic salmon pineal gland are thermo-receptors. Other functions are, however, not excluded, in keeping with the observation that TRPV channels are multimodal channels as commented above. Indeed, the observation that most of the ISH detected TRPV channels were located in the apical part of the photoreceptors, bathing into the CSF, in the Atlantic salmon pineal gland would agree with pioneer studies suggesting the pineal organ of ectotherms is involved in the regulation of pressure or composition of the CSF (Kelly and Vandekamer, 1960). This is particularly relevant in the Atlantic salmon, a migratory species, in which the life cycle involves adaptation to waters of different salinities, and migration start is triggered by changes in both photoperiod and temperature. In line with this, we found TRPV1 and TRPV4 channels in the apex of *saccus dorsalis* cells, which plays a major role in the production and regulation of the CSF in trout (Jansen et al., 1976a,b). The *saccus dorsalis* is apparently an analog of the choroid plexus (absent in trout) and is involved in a number of functions including fluid secretion, catabolism and extrusion of organic substances (monoamines, GABA) into the ventricular system, and uptake of organic substances from the CSF. It is interesting that the pineal gland and the *saccus dorsalis* of ectotherms, both receive innervation from arginine vasotocin fibers originating from the preoptic area (Vandendungen et al., 1982; Ramallo et al., 2012). Arginine vasotocin is a regulator of water balance and osmotic homoeostasis.

### TRPV1 and TRPV4 in the Retina

The cellular localization of TRPV1 and TRPV4 in the retina of *S. salar* was performed using IHC only (preliminary investigations using ISH indicated both, ISH and IHC, provided similar results; data not shown). We felt interesting to investigate the localization of TRPV1 and TRPV4 in the salmon retina for several reasons: (i) the fish pineal organ and retina are two homologous organs, (ii) previous studies indicated that TRPV channels are present in the vertebrates’ retina (Gao et al., 2019; Toft-Bertelsen et al., 2019; Bouskila et al., 2020) but (iii) data on fish remain scarce and concern the retina of 3 species only, namely *D. rerio* (Zimov and Yazulla, 2004; Amato et al., 2012; Sanchez-Ramos et al., 2012), *C. auratus* (Zimov and Yazulla, 2004), and *O. mykiss* (Nisembaum et al., 2015). Major differences between the pineal organ and the retina lie in the facts that the former contains only cones and does not possess a complex network of interneurons between the photoreceptors and ganglion cells. Also, the pineal is an “open organ,” while the retina is a “closed organ,” in other words, at their apical parts, the inner and outer segments of the photoreceptors bath into the CSF in the pineal gland, while those of the retina are nested into the extensions of the retinal pigment epithelial cells; at their basal part, the pineal organ is opened to the blood circulation, being directly surrounded by vessels, while the retina bathes into the vitreous humor.

In a general manner, Atlantic salmon TRPV1- and TRPV4-like proteins distributed as described in other vertebrate species [*fish*: (Sanchez-Ramos et al., 2012; Nisembaum et al., 2015); *mouse*: (Ryskamp et al., 2011; Lakk et al., 2018); *Monkeys and human*: (Gau et al., 2013; Sappington et al., 2015)]. Most of the studies dealing with TRPV1 and TRPV4 in the vertebrates’ retina indicate a role in detecting variations in temperature, osmotic pressure, mechanical, volume and hydrostatic changes (linked to systemic changes in blood pressure, hydrostatic pressure from the CSF and intrinsic intraocular pressure), as well as in mediating the response to chemicals (lipids and endocannabinoids) (Alessandri-Haber et al., 2006; Ryskamp et al., 2011, 2014; Ye et al., 2012; Sappington et al., 2015; Toft-Bertelsen et al., 2019; Pang et al., 2021; Redmon et al., 2021). In the Atlantic salmon retina, TRPV1 and TRPV4 were expressed in some, but not all, photoreceptors, highlighting a heterogeneity among the photoreceptor cell types; this contrasts with the situation observed in the pineal gland of this same species, or the retina of the rainbow trout, in which all the ONL appeared to express TRPV1 and TRPV4. These differences observed from a study to another might be due to either species-specific requirements or to differences in the experimental protocols (e.g., time of day or season) and technical approaches (e.g., ICC vs. ISH), not mentioning that TRPV mRNA expression may change from eye to eye, as reported to occur for TRPV1 in mice (Sappington et al., 2015). Whether these channels contribute to controlling melatonin production by the retinal photoreceptors, as is the case for their pineal analogs, remains an open question. It is also possible that they contribute to modulate neural transmission to bipolar cells, as they have been localized associated to the photoreceptor synaptic ribbons in *D. rerio* and *C. auratus* (Zimov and Yazulla, 2004), and to mediate synaptic transmission in rod bipolar cells in mice (Shen et al., 2009). Another interesting possibility is that TRPV channels contribute to controlling the temperature driven shifts between rhodopsin and porphyropsin observed in various fish species (including salmonids) and which affects nocturnal spectral sensitivity (Cristy, 1976; Saszik and Bilotta, 1999; Flamarique, 2005).

## Conclusion

In fish, the pineal organ is part of the thermo-receptive circuitry together with other key temperature-sensitive neurons located in the brain, spinal cord and lateral line (Rubin, 1934; Greer and Gardiner, 1970, 1974; Iriki et al., 1976; Nagai et al., 1977; Haesemeyer, 2020). The present study in the Atlantic salmon brings novel information concerning the distribution of the thermo-sensitive TRPV1 and TRPV4 channels, particularly in the photosensitive pineal gland and retina. We extend to *S. salar* data obtained in another salmonid, *O. mykiss*: i.e., the channels are specifically expressed in the cone photoreceptors of the fish pineal gland, where they contribute to controlling the nocturnal rise in melatonin secretion, the hormonal time-keeper in vertebrates (Nisembaum et al., 2015). In the rainbow trout, TRPV1 activation is temperature dependent. Given the similarities in the *in vitro* impacts of TRPV analogs on melatonin production by rainbow trout and Atlantic salmon pineal glands, it is reasonable to believe that TRPV channels also respond to temperature in *S. salar* pineal gland, supporting previous conclusions that the pineal photoreceptor is a “photo-thermo-receptor” (Falcón et al., 2007; Nisembaum et al., 2015). Light, through controlling the VGCC (Falcón et al., 2007), and temperature through the TRPV1 and TRPV4 channels, both appear to modulate melatonin secretion *via* the control of Ca^2+^ entry within the photoreceptor cells (see discussion in Nisembaum et al., 2015). A similar pathway might also be controlling the release of the excitatory neurotransmitter at the synaptic junction between photoreceptor cells and ganglion cells, as the electrical activity of the latter is also light- and temperature-dependent (Tabata and Meissl, 1993; Tabata et al., 1993). Future functional studies in the pineal gland of fish should shed light on the exact role played by TRPV1 and TRPV4 channels as thermo-sensors, but also as volume and osmotic sensors.

In the context of the ongoing global changes, more investigations are urgently needed to further elucidate the roles of TRPV in the pineal and retinal physiology of the Atlantic salmon, and more generally to elucidate how the fish senses temperature. Indeed, the salmon of the Loire/Allier basin is an endangered species, which like other Atlantic salmon populations, is experiencing a continuous decline since the early twentieth century, due to the impact of a series of factors including a rise in temperature (Thibault, 1994; Parrish et al., 1998; Limburg and Waldman, 2009; Zhang, 2017). In the past three decades the waters of the Loire/Allier increased by ∼2°C (Gosse et al., 2008; Marschall et al., 2011; Martin et al., 2012) and another + 4°C increase is predicted for the end of this century (Moatar et al., 2010). Unraveling the mechanisms of thermo-reception and thermo-regulation in fish becomes crucial in order to anticipate the impacts of the current temperature changes.

## Data Availability Statement

The datasets presented in this study can be found in online repositories. The names of the repository/repositories and accession number(s) can be found in the article/Supplementary Material.

## Ethics Statement

The animal study was reviewed and approved by “Ethics Committee for Animal Experiment of Languedoc-Roussillon (C2EA-LR/C2EA-36)” N° A6601601, and the European Union regulations (European directive 2010/63/EU).

## Author Contributions

LGN: performed experiments (HPLC, ISH, IHC, organ culture, qPCR), supervision of students, data analysis, and manuscript writing. GL and TL’H: performed experiments and manuscript reading. PM: experimental design, infrastructures and personnel supervision, performed experiments, and manuscript reading. MF: technical assistance (sampling). C-HP: technical assistance (organ culture, ISH, IHC). KE: technical assistance (HPLC). MJD: funding, supervision of post-doc, and manuscript reading. LB: performed experiments (ISH, IHC), supervision of students, and manuscript reading. JF: funding, experimental design, data analysis, writing, supervision of students, and performed experiments. All authors contributed to the article and approved the submitted version.

## Conflict of Interest

The authors declare that the research was conducted in the absence of any commercial or financial relationships that could be construed as a potential conflict of interest.

## Publisher’s Note

All claims expressed in this article are solely those of the authors and do not necessarily represent those of their affiliated organizations, or those of the publisher, the editors and the reviewers. Any product that may be evaluated in this article, or claim that may be made by its manufacturer, is not guaranteed or endorsed by the publisher.

## Abbreviations

4αPDD: 4α-Phorbol 12,13-didecanoate
AANAT: arylalkylamine *N*-acetyltransferase
cAMP: cyclic AMP
CSF: cerebrospinal fluid
GCL: ganglion cell layer
IHC: immunohistochemistry
INL: inner nuclear layer
IPL: inner plexiform layer
ISH: *in situ* hybridization
ONL: outer nuclear layer
OPL: outer plexiform layer
PFA: paraformaldehyde
TRPV: transient receptor potential channels vanilloid
VGCC: voltage-gated Ca^2+^ channels.
