# Supplementary material for: Transient Receptor Potential-Vanilloid (TRPV1-TRPV4) Channels in the Atlantic Salmon, Salmo salar. A Focus on the Pineal Gland and Melatonin Production
Source: Front Physiol. 2022 Jan 7;12:784416. doi: 10.3389/fphys.2021.784416 (PMC8782258; doi:10.3389/fphys.2021.784416)
Supplement: Supplementary file 1 [file Data_Sheet_1.docx]

**Transient Receptor Potential-vanilloid (TRPV1-TRPV4) channels in the Atlantic salmon, *Salmo salar*. A focus on the pineal gland and melatonin production**

Laura Gabriela Nisembaum, Guillaume Loentgen, Thibaut L’Honoré, Patrick Martin, Charles-Hubert Paulin, Michael Fuentès_,_ Karine Escoubeyrou, María Jesús Delgado, Laurence Besseau, Jack Falcón

### SUPPLEMENTARY MATERIAL

**Supplementary Figure 1 – Alignment of the amino acid sequences from Salmo salar TRPV1 (NP_001133970.1)** **and TRPV4** (**XP_014016244.1).**

Alignment was performed using the BLAST tool from NCBI. The two sequences display 49% identity (*), and 65% strong (:) or weak (.) similarity. The colored aa residues correspond to the sequences amplified by the qPCR (red) and ISH (purple) primers. These sequences displayed less than 60% identity

TRPV1 ------------------------------------------------------------

TRPV4 MNEDRSPATLLRRCRIAMTETDTLHSDANKAALSSGSGEGGGSGEGQPDADGTCPDLSAL

TRPV1 ---MSKSKGPEYPSFSLETDDRTDDERAQSRQVKKPDRLVSALGLGSSGGPKTPMDSDYQ

TRPV4 ADLFESEEGSQSPQDPAPDVDRPGQLQPGDSRQNLRMKFHGAFKKGISNPMDLLESTIYE

:...:*.: *. . **..: :. . : : :: .*: * *. . .: *:

TRPV1 DELEEAAPKIRFNLNFDKEVRCLEENKEDR--------------------------VSKR

TRPV4 SPVAPGPKKAPMDSLFDYGTYRHTNNKKPRRKKLPRGKTETSCNESLDPPGLDPPKVLKV

. : .. * :: ** . :**: * * *

TRPV1 FDIKRLFEAVSTGDVMKLEGLHQYLHQSMKKLSNTEYQS--YGKN**VLLKALLNLRKGRNN**

TRPV4 FNRMLLFDGVSRADPEALSGLLEYLQGHEKRLTDEEFKEPSTGKTCLPKALLNLYSGQND

*: **:.** .* *.** :**: *:*:: *::. **. * ****** .*:*:

TRPV1 **TIEYLLDISEKMGDIKEFVNAAYTD**SYYKGQTALHIAIERRSIYFVELLIKKGANVHAKA

TRPV4 TIPMLMDIAEQTVNLHEFINTPFRDVYYRGQTALHIAIERRCKQYVELLVEKGADVHAQA

** *:**:*: :::**:*:.: * **:************. :****::***:***:*

TRPV1 CGKFFQAHD-GPSFYFGELPLSLAACTNQPEVVDFLLENDYQRVDVRESDSLGNMVLHAL

TRPV4 RGRFFQPRDEGGYFYF**GELPLSLAACTNQPNMVHYLTENAHKKADLRRQDSRGNTVLHAL**

*:***.:* * *****************::*.:* ** :::.*:*..** ** *****

TRPV1 VVLADNTPENTDFITSMYDHILTTTARLHPEWRLEDIENNQGLTTIKLAAKTGKIGLFKH

TRPV4 **VHIADNTRDNTRFLTKMYDLLLTKCAKLYPECSLEDILNNDGMSPLMMAAKLGKIG**VFQH

* :**** :** *:*.*** :**. *:*:** **** **:*::.: :*** ****:*:*

TRPV1 MMHREFQERETRHLSRKFTEWVYGPVHSSLYDLASLDSY-EKNSVMEIIVYSSDIPNRHE

TRPV4 IIRREIKDEEARHLSRKFKDWAYGPVYSSLYDLSSLDTCGEEVSVLEILVYNSRI**ENRHE**

:::**:::.*:*******.:*.****:******:***: *: **:**:**.* * ****

TRPV1 MLQIEPLNRLLEEKWDKFAARMFFLNFLVYLVYLSVFTAVAYNRKKGTPPFTLEHTRQEY

TRPV4 **MLAVEPINELLRVKWQKFAAVTFYISVVSYLVTMIIFTLVAYYRPS**QGMPPYPYTTSTDY

** :**:*.**. **:**** *::..: *** : :** *** * . * * :*

TRPV1 LRLAGQLFITVGACYFFIRGILDLKRKRPS-LDTLLIDGYSEILFFLQAIFFLASLVLYC

TRPV4 LRLGGEVITLGSGVFFFLTNIKDLFLKKCPGVNSLFVDGSFQLLYFIYSVLVIVTAALYL

***.*::: .. :**: .* ** *: . :::*::** ::*:*: :::.:.: .**

TRPV1 CGREEYLGFLVLCLALSWVNLLYFSRGYRHMGIYSVMIQKMILSDILRFLFVYVTFLFGF

TRPV4 SGIEAYVSVMVFALVLGWMNTLYFTRGLKLTGTYSIMIQKILFKDLFRFLLVYVLFMIGY

.* * *:..:*:.*.*.*:* ***:** : * **:****:::.*::***:*** *::*:

TRPV1 SAAVVTLLMEPELPASNTAQPINSTDGKGRTLFLPTEDSCIKPTFRNISHTIMELFKFTI

TRPV4 SSALVSLLAVCPGPDEVCPEEG------------GCPTYPQCRDTDTFSNFLLDLFKLTI

*:*:*:** * . .: .:*: :::***:**

TRPV1 GMGDLEFTEGYQYKEVFYMLLISYIVLTYILLLNMLIALMSRTVEKMSLEST**SIWKLQRA**

TRPV4 GMGDLDMVSSAQYPAVFLILLVTYIILTFVLLLNMLIALMGETVSQVSKESKKIWKLQWA

*****::... ** ** :**::**:**::**********..**.::* **..***** *

TRPV1 **ITILDLERSLPRCLRRRLRSGVDKDLGTRAG-EKDRRWCFRVEEVNWNKWNTNLGIINED**

TRPV4 TTILDIERSFPVCLRKSFRSGEMVTVGKNWDGTPDRRWCFRVDEVNWCHWNQNLAIINED

****:***:* ***: :*** :*.. . ********:**** :** **.*****

TRPV1 **PGSGDTARLSPSHSSRTLGKERSWRGFLGNVSRRQHTQPQHQIQVESTEMSSLSPLSHV**-

TRPV4 PGKNITETQQCSGTVHQTVRGLRRDRWSTVVPRVVEQNKGPRPRDLVLEMEPLTPRHRPC

**.. * . * : : : : *.* . : : : **..*:* :

TRPV1 --- 804

TRPV4 AEG 891

**Supplementary Figure 2. Alignment of Atlantic salmon, *Salmo salar* TRPV1 amino acid sequence (NP_001133970.1)** **with the corresponding sequences from other vertebrates.** The domains were determined using InterProScan. The blue lines mark the ankyrin repeat containing domains; the aa residues from the ankyrin repeats are in red. The dotted green lines correspond to the channel domains, with the corresponding transmembrane domains (S1 to S6 from top to the bottom) delimited by the red squares. Asterisk, fully conserved residues; column, residues displaying strongly similar properties; period, residues with weak similar properties. Clustal Omega (CLUSTAL O (1.2.4) multiple sequence alignment). *Oncorhynchus mykiss* (AIZ00833.1), *Danio rerio* (NP_001119871.1), *Carassius auratu*s (XP_026106568.1), *Oryzias latipes* (XP_011482044.1), *Xenopus laevis* (ADE62146.1), *Gallus gallus* (NP_989903.1), *Homo sapiens* (NP_542436.2) and *Rattus norvegicus* (NP_114188.1). Amino acid residues, which roles have been identified in the rat (so far the best characterized sequence among the TRP) are highlighted in color/bold. Y653 has been associated to temperature gating; this residue is conserved in all the fish sequences displayed. The residues **N628** and **N652** located between the transmembrane domains 5-6 (*i.e*., the pore region) mediate temperature sensitivity; none is conserved in *S. salar* and other teleost sequences. Q727 and W752 located in the C-terminal end of the sequence are believed to be important for transmitting the temperature response; in fish the glutamine residue is replaced by residues with similar properties, while the tryptophan residue is conserved. Residues E600 and E648 mediate the acidic activation of the channel; E648 is conserved in salmonids and other fish species. The residues S512 and T550 of the rat sequence are important for the response to capsaicin, only serine has been conserved in the salmonids sequences, none is present in the other represented teleost (zebrafish was demonstrated to not respond to capsaicin). **Y511**, **M547** and **T551** are anandamide binding sites; while tyrosine has been preserved among vertebrates, methionine has been replaced by leucine (similar properties) in human, chicken, salmon and other fish, and threonine has been replaced by valine or isoleucine (no equivalent properties). The two S502 and S800 serine residues are identified as phospholipase C phosphorylation sites that might contribute to channel sensitization; both are conserved in salmonids. Residue K710 is important for the response to lysophosphatidic acid (LPA). (For details on the TRPV1 molecular structure see: Leonelli et al., 2011; Cohen and Moiseenkova-Bell, 2014; Cordero-Morales and Vasquez, 2018; Shuba, 2020).

**TRPV1 Protein Alignment**

X.laevis -------MKKMGSSTDIDETEETCASIETDESHSDDTNRSAQENRKKLKFCQAKYSIFSS 53

G.gallus MSSILEKMKKFGS-SDIEESEVTDEHTDGEDSALETADN--LQGTFSNKVQPS-KSNIFA 56

H.sapiens -------MKKWSS-TDLGAAADPLQ----KDTCP---DP--LDGDPNSRPPPA-KPQLST 42

R.norvegicus -------MEQRAS-LDSEESESPPQ----ENSCL---DP--PDRDPNCKPPPV-KPHIFT 42

O.latipes ----------------MRKSEMFGFSLETDDRTKE----------ELLRDKPTKKGV--- 31

**S.salar** -------------MSKSKGPEYPSFSLETDDRTDD----------ERAQSRQVKKPD--- 34

O.mykiss -------------MSKSKGPEYPSFSLETDDRTDD----------ERAQSRQVKKPD--- 34

D.rerio -----------------MDSSVSSFSLETDDQTEE----------ERTKAKQMKK----- 28

C.auratus --------------MSTSEDSSPTFFLETDDLTDE----------ERSKFKQVKKG---- 32

**.: :**

X.laevis PKPKGRRFGKTETDRDMAPMDSVYQIESKV------ISPAIKFHRNLERGKLCNQLVRQS 107

G.gallus RRGR---FVMGDCDKDMAPMDSFYQMDHLM------APSVIKFHANMERGKLHKL----- 102

H.sapiens AKSRTRLFGKGDS-EEAFPVDCPHEEGELDSCPTITVSPVITIQRPGDGPTGARL----- 96

R.norvegicus TRSRTRLFGKGDS-EEASPLDCPYEEGGLASCPIITVSSVLTIQRPGDGPASVRP----- 96

O.latipes --DFLRG-----QETAEPPMDTDYHEEKEKP------APQLRFNLGFDKLARGKE--QNK 76

**S.salar** --RLVSALGLGSSGGPKTPMDSDYQDELEEA------APKIRFNLNFDKEVRCLE--ENK 84

O.mykiss --RLVSALGLGSSGGPKAPMDSDYQDELEEA------APKIRFNLYFDTEVRCLE--ENK 84

D.rerio ------------VSKDKRPMDSNYVDDVVEP------SSTIKFNLHFDRGIRNLK--EEP 68

C.auratus ------------TSKDKRPMDSDYLEETDDS------AHTIKFNLNFDGGIRNVK--EEP 72

***:* : : :: :**

X.laevis TSLESTSSCKDRTFKLYDQRRIFDAAAYGDCEELDDLLVYLLRTHKRLTNEEFKEKETGK 167

G.gallus LSTDSITGCSEKAFKFYDRRRIFDAVARGSTKDLDDLLLYLNRTLKHLTDDEFKEPETGK 162

H.sapiens LSQDSVAASTEKTLRLYDRRSIFEAVAQNNCQDLESLLLFLQKSKKHLTDNEFKDPETGK 156

R.norvegicus SSQDSVSA-GEKPPRLYDRRSIFDAVAQSNCQELESLLPFLQRSKKRLTDSEFKDPETGK 155

O.latipes R--------D---TRFTR-DLLFEAAASGDVQKLEGLEDYLRLNMKNLSDSLYQS--YGK 122

**S.salar** E--------DRVSKRFDI-KRLFEAVSTGDVMKLEGLHQYLHQSMKKLSNTEYQS--YGK 133

O.mykiss E--------DRDSKRFDIIKRLFEAVSTGDVMKLEGLHQYLHQSMKKLSNTEYQS--YGK 134

D.rerio A--------QQDNDRFTI-KRLFEAVSSGDVSKMQGLHEYLHKNMKRLTDSQYKS--NGK 117

C.auratus A--------QQDRERFTL-KRLFDAVSSGNVSKLQGLHEYLHKNMKRLTDSEYKS--NGK 121

**:: :*:*.: .. .::.* :* . *.*:: ::. ****

X.laevis TCLLKAMLNLDKGMNHTILLFLEIAEKTDNLKEFINSAYRDNYYRGQTALHIAIERRNMD 227

G.gallus TCLLKAMLNLHDGKNDTIPLLLDIAKKTGTLKEFVNAEYTDNYYKGQTALHIAIERRNMY 222

H.sapiens TCLLKAMLNLHDGQNTTIPLLLEIARQTDSLKELVNASYTDSYYKGQTALHIAIERRNMA 216

R.norvegicus TCLLKAMLNLHNGQNDTIALLLDVARKTDSLKQFVNASYTDSYYKGQTALHIAIERRNMT 215

O.latipes TPLIKALMHLKDGKNKTVEIFINTAKNIGDLEKFVNAAHTSNYYKGQTALHVAIERRSLP 182

**S.salar** NVLLKALLNLRKGRNNTIEYLLDISEKMGDIKEFVNAAYTDSYYKGQTALHIAIERRSIY 193

O.mykiss NVLLKALLNLRNGRNNTIEYLLDISEKMGDINELVNAAYTDSYYKGQTALHIAIERRSTY 194

D.rerio TALLKALLNLRQGENDTIEQLLDIAEKMGDLKNFINAAYTDSYYKGQTALHVAIERRSMK 177

C.auratus TALLKALLNLKEGENDTIELLLEIAEKTGGLKSLVNAAYTDIYYKGQTALHVAIERRSAK 181

**. *:**:::* .* * *: ::: :.: . ::.::*: : . **:******:*****.**

X.laevis LVQLLLQHGADVHARADGEFFRKAKGKAGFYFGELPLSLAACTNQTAIVRYLLQNQHSPA 287

G.gallus LVKLLVQNGADVHARACGEFFRKIKGKPGFYFGELPLSLAACTNQLCIVKFLLENPYQAA 282

H.sapiens LVTLLVENGADVQAAAHGDFFKKTKGRPGFYFGELPLSLAACTNQLGIVKFLLQNSWQTA 276

R.norvegicus LVTLLVENGADVQAAANGDFFKKTKGRPGFYFGELPLSLAACTNQLAIVKFLLQNSWQPA 275

O.latipes YVQLLVNSHADVHAKVSGKFFQPHD-GPCFYFGELPLSLAACTNQPEMVDYLLKNEIQRA 241

**S.salar** FVELLIKKGANVHAKACGKFFQAHD-GPSFYFGELPLSLAACTNQPEVVDFLLENDYQRV 252

O.mykiss FVELLIKKGANVHAKACGKFFQLND-GPSFYFGELPLSLAACTNQPEVVDFLLENDYHRV 253

D.rerio FVQMLVKKGADVHAKACGKFFQPNQ-KMCFYFGELPLSLAACTNQQDIVDFLMENPHQAV 236

C.auratus FVKMLVEKGADVHAKACGKFFQPNQ-EACFYFGELPLSLAACTNQPDIVDFLMDNPYKRV 240

*** :*:: *:*:* . *.**: . **************** :* :*:.* .**

X.laevis NIAARDSFGNTVLHALVDIADNTQENTAFVTKMYNEILVLGAQIKPSLKIEEIANKKGLT 347

G.gallus DIAAEDSMGNMVLHTLVEIADNTKDNTKFVTKMYNNILILGAKINPILKLEELTNKKGLT 342

H.sapiens DISARDSVGNTVLHALVEVADNTADNTKFVTSMYNEILMLGAKLHPTLKLEELTNKKGMT 336

R.norvegicus DISARDSVGNTVLHALVEVADNTVDNTKFVTSMYNEILILGAKLHPTLKLEEITNRKGLT 335

O.latipes DPEQRDSHGNTVLHALVAVADNSKENTEFINSMYDRILKITAKLHPKKKLEDIKNNKGLS 301

**S.salar** DVRESDSLGNMVLHALVVLADNTPENTDFITSMYDHILTTTARLHPEWRLEDIENNQGLT 312

O.mykiss DVRESDSLGNMVLHALVVLADNTPENTDFITSMYDHILTTAARLHPKWRLEDIENNQGLT 313

D.rerio DVRERDCHGNTVLHALVSVADNSPENTEFVIAMYDHILIKADQLHPKTKLEEIENNEGLT 296

C.auratus DVGNRDSHGNTVLHALVSIADNSPENTEFVIAMYDHILLKANQLHPEIKLEEIKNNKGLT 300

**: *. ** ***:** :***: :** *: **:.** :::* ::*:: *.:*::**

X.laevis PLSLAAKTGKIGVFAYILRREIKNLECRHLSRKFTEWAYGPVHSSLYDLSGVDTYEKNSV 407

G.gallus PLTLAAKTGKIGIFAYILRREIKDPECRHLSRKFTEWAYGPVHSSLYDLSCIDTCEKNSV 402

H.sapiens PLALAAGTGKIGVLAYILQREIQEPECRHLSRKFTEWAYGPVHSSLYDLSCIDTCEKNSV 396

R.norvegicus PLALAASSGKIGVLAYILQREIHEPECRHLSRKFTEWAYGPVHSSLYDLSCIDTCEKNSV 395

O.latipes PLLMAAKTGKIGVFSHILKREFHESDIKHLSRKFTEWVYGPVHCSLYDLASVDSCENNSL 361

**S.salar** TIKLAAKTGKIGLFKHMMHREFQERETRHLSRKFTEWVYGPVHSSLYDLASLDSYEKNSV 372

O.mykiss TIKLAAKTGKIGLFKHMMHREFQERETRNLSRKFTEWVYGPVHSSLYDLDSLDSYEKNSV 373

D.rerio PITLAAKKGKLGLFKHIVQRELM--GCRHLSRKITEWAYGPVCSSLYDLSSLDTYEKNSA 354

C.auratus PLTLAAKTGKVVLLKHIVQREFK--GCKHLSRKITEWAYGPVCSSLYDLSSLDTYEKNSA 358

**: :** .**: :: ::::**: ::****:***.**** .***** :*: *:****

X.laevis LEIIAYS-SETPNRHDMLLVEPLNKLLQDKWDHFVKRIFYFNFFAYIIYVIIFTIAAYYR 466

G.gallus LEIIAYS-SETPNRHEMLLVEPLNRLLQDKWDRFVKHLFYFNFFVYAIHISILTTAAYYR 461

H.sapiens LEVIAYSSSETPNRHDMLLVEPLNRLLQDKWDRFVKRIFYFNFLVYCLYMIIFTMAAYYR 456

R.norvegicus LEVIAYSSSETPNRHDMLLVEPLNRLLQDKWDRFVKRIFYFNFFVYCLYMIIFTAAAYYR 455

O.latipes LEILIYG-SDIPNRHEMLQTEPLSQMLQAKWKKFAGGMFLINFLVYSLYLTIFTFVAHYI 420

**S.salar** MEIIVYS-SDIPNRHEMLQIEPLNRLLEEKWDKFAARMFFLNFLVYLVYLSVFTAVAYNR 431

O.mykiss LEIIVYS-SEIPNRHEMLQIEPLNRLLEEKWDKFAARMFFLNFLVYLVYLSVFTAVAYYR 432

D.rerio LEIVVYG-SEIPNRLEMLQIEPLNRLIEEKWDQFAHRMFLFNFIVYVIYLFIFTASAFYH 413

C.auratus LEIIVYG-SEIPNRLEMLNIEPFNRLIEEKWERFAKRMFLFSFIVYVIYLFIFTAVAYNR 417

**:*:: *. *: *** :** **:.:::: **.:*. :* :.*:.* ::: ::* *.**

X.laevis PVD-------GSPPFPVQ--YGSYLRTSGELITVIGGIYFFFRAIQYFTQRRP**S**LKALLA 517

G.gallus PVQKG-----DKPPFAFGHSTGEYFRVTGEILSVLGGLYFFFRGIQYFVQRRP**S**LKTLIV 516

H.sapiens PVD-------GLPPFKMEK-TGDYFRVTGEILSVLGGVYFFFRGIQYFLQRRP**S**MKTLFV 508

R.norvegicus PVE-------GLPPYKLKNTVGDYFRVTGEILSVSGGVYFFFRGIQYFLQRRP**S**LKSLFV 508

O.latipes RGTAEYREFPF----P-LKSYDDYLFATGLLLTLLANLFLFIAGITDMWRKRPNMMTVLI 475

**S.salar** KKGTP--------PFTLEHTRQEYLRLAGQLFITVGACYFFIRGILDLKRKRP**S**LDTLLI 483

O.mykiss KKGTP--------PFTLEHTRQEYLRLAGQLFITVGACYFFIRGILDLKRXRP**S**LDTLLI 484

D.rerio EEGKDYANQP---PYLYAKSREGYLLLTGHIISITGAFYFFIRGLIDMVRKRPRFQSLII 470

C.auratus EEEKDFSNKTLKSSLRYKNNSKGYLLLIGQIITTIGALYFLIKGLIDMLRKRPGFQSLFI 477

***: * :: . :::: .: : : ** : :::**

X.laevis DS**Y**CEFLFFSQSVFLLLSTVLYFCGRNEYVAFLVICLA**M**SWANVLYYTRGFQLMGIYSVM 577

G.gallus DS**YS**EVLFFVHSLLLLSSVVLYFCGQELYVASMVFSLA**L**GWANMLYYTRGFQQMGIYSVM 576

H.sapiens DS**YS**EMLFFLQSLFMLATVVLYFSHLKEYVASMVFSLA**L**GW**T**NMLYYTRGFQQMGIYAVM 568

R.norvegicus DS**YS**EILFFVQSLFMLVSVVLYFSQRKEYVASMVFSLA**M**GW**T**NMLYYTRGFQQMGIYAVM 568

O.latipes DG**Y**YEILFCVQGLFYLCFAVLYVAGLKEYVCFLVLCLA**L**SWVNVLYFSRGYQHMGIYSVM 535

**S.salar** DG**YS**EILFFLQAIFFLASLVLYCCGREEYLGFLVLCLA**L**SWVNLLYFSRGYRHMGIYSVM 543

O.mykiss DG**YS**EILFFLQAIFFLASSVLYCCGREEYLGFFVLCLA**L**SWVNLLYFSRGYRHMGIYSVM 544

D.rerio DG**Y**TDQLFFVQGLLFLASVVLYCYGQYEYLAFLVLCLA**L**SWINLLYFSRGSKNLGIYNVM 530

C.auratus DG**Y**TDQLFFLQAVLFLACALLYFFGQDEYVACLVLCLA**L**SWVNLLYFSRGSKNMGIYNVM 537

***.* : ** :.:: * :** *: :*:.**:.* *:**::** : :*** ****

0 D-rerio_TRPV1-1

X.laevis IEKLILSDMVRFLFVYLLFLFGFAAALVTLI**E**DGEGRTDVNN------------------ 619

G.gallus IAKMILRDLCRFMFVYLVFLLGFSTAVVTLI**E**DDNEGQDTNS------------------ 618

H.sapiens IEKMILRDLCRFMFVYIVFLFGFSTAVVTLI**E**DGKNDSLPSE------------------ 610

R.norvegicus IEKMILRDLCRFMFVYLVFLFGFSTAVVTLI**E**DGKNNSLPME------------------ 610

O.latipes IQKIIVCDILRFLFVYVVFLFGFSAAVVTLLIVPPEKPRN-------------------- 575

**S.salar** IQKMILSDILRFLFVYVTFLFGFSAAVVTLLMEPELPASN-------------------- 583

O.mykiss IQKMILCDILRFLFVYVTFLFGFSAAVVTLLMEPELPDNN-------------------- 584

D.rerio IQKMVLGEIRRFLVVYMVFLIGFSAALVTLLDQESIDSGSTRDFRLSEDIPSLNPTPDSS 590

C.auratus IQKMVLGEIRRFLVVYMVFLIGFSTAVVTLLDEGPISAQS-------------------- 577

*** *::: :: **:.**: **:**::*:***:**

0 D-rerio_TRPV1-1

0 D-rerio_TRPV1-1

X.laevis -----------------T--------CGRRCCKPEPASY**N**NLYYTCQELFKFAIGMGDL**E** 654

G.gallus -------------SEYA----------RCSHTKRGRTSY**N**SLYYTCLELFKFTIGMGDL**E** 655

H.sapiens -------------STSHR--------WRGPACRPPDSSY**N**SLYSTCLELFKFTIGMGDL**E** 649

R.norvegicus -------------STPHK--------CRGSACKPG-NSY**N**SLYSTCLELFKFTIGMGDL**E** 648

O.latipes ---------------ATKGRSFFTTQAPGEDCF--IPTYKNFSFTVLELFKFTIGMGDM**E** 618

**S.salar** -------TAQPINSTDGKGRTLFL--PTEDSCI--KPTFRNISHTIMELFKFTIGMGDL**E** 632

O.mykiss -------TAQPINSTDGRGRTFFL--PTEDSCI--KPTFRNISHTIMELFKFTIGMGDL**E** 633

D.rerio NPQSRMTHHQPTTARDGRGRFGLTTDNQYEVCK--KPSYKNIYFTTLELFKFTIGMGDL**E** 648

C.auratus --------------------------SSEGHCT--KPSFKSIYYTTLELFKFTIGMGDL**E** 609

**::..: * *****:*****:***

X.laevis FTD**NY**KYKPVFIFLLITYVILTYILLLNMLIALMGETVSKVAQESKSIWKLQRAITILDI 714

0 D-rerio_TRPV1-1

G.gallus FTE**NY**RFKSVFVILLVLYVILTYILLLNMLIALMGETVSKIAQESKSIWKLQRAITILDI 715

H.sapiens FTE**NY**DFKAVFIILLLAYVILTYILLLNMLIALMGETVNKIAQESKNIWKLQRAITILDT 709

R.norvegicus FTE**NY**DFKAVFIILLLAYVILTYILLLNMLIALMGETVNKIAQESKNIWKLQRAITILDT 708

O.latipes FSQAFQYTEIFYFLLIGYIILTYILLLNMLIALMNRTVENITKESTCIWKLQRAVTILDM 678

**S.salar** FTEG**Y**QYKEVFYMLLISYIVLTYILLLNMLIALMSRTVEKMSLESTSIWKLQRAITILDL 692

O.mykiss FTEG**Y**QYKEVFYMLLISYIVLTYILLLNMLIALMSRTVEKMSLESTSIWKLQRAITILDL 693

D.rerio FTDH**Y**KYKEVFYVLLIVYIVMTYILMLNMLIALMNQSVEMMSVESTSIWKLQRAITTLDM 708

C.auratus FTDQFQYIEVFYVLLILYIVMTYILMLNMLIALMNQRVEEMSVESTSIWKLQRAITTLDM 669

***:: : : :* .**: *:::****:********.. *. :: **. *******:* ****

X.laevis E**K**SFLNSFRDTFRSGKSVLVGITPDGKEDYRWCFRVDEVNWNK**W**NSNLGII-KEDPGNCH 773

G.gallus ENSYLNCLRRSFRSGKRVLVGITPDGQDDYRWCFRVDEVNWST**W**NTNLGII-NEDPGCSG 774

H.sapiens E**K**SFLKCMRKAFRSGKLL**Q**VGYTPDGKDDYRWCFRVDEVNWTT**W**NTNVGII-NEDPGNCE 768

R.norvegicus E**K**SFLKCMRKAFRSGKLL**Q**VGFTPDGKDDYRWCFRVDEVNWTT**W**NTNVGII-NEDPGNCE 767

O.latipes E**K**RLPYCLRKRLRCGVEKKLCTA--LGNDQRWCFRVEEVNWNK**W**NTDIGKI-DEDPGYYD 735

**S.salar** E**R**SLPRCLRRRLRSGVDKDLGTRA-GEKDRRWCFRVEEVNWNK**W**NTNLGII-NEDPGSGD 750

O.mykiss E**R**SLPRCLRRRLRSGVDKDLGTRA-GEKDRRWCFRVEEVNWNK**W**NTNLGII-NEDPGSGD 751

D.rerio EWILPKCLQGKLRSGEEKDLGGG--QEPDRRWCFSVEEVNWTQ**W**NRNMGIIINEDPGKCT 766

C.auratus EWILPRCLKTKLRSGEEKDLGGE--QEPDRRWCFSVEEVNWNV**W**NRNLGVV-TEDPGKCI 726

*** .:: :*.* : * **** *:****. ** ::* : ******

X.laevis GFKSTLSASFRP---RGRRWRS--LVPHIKEINLGNENE-TVPEEVPLQIQPALSVQTVK 827

G.gallus DLKRNPSYCIKPGRVSGKNWKT--LVPLLRDG**S**RREETP-KLPEEIKLKPILEPY---YE 828

H.sapiens GVKRTLSFSLRSSRVSGRHWKNFALVPLLREA**S**ARDRQS-AQPEEVYLRQFSG-S---LK 823

R.norvegicus GVKRTLSFSLRSGRVSGRNWKNFALVPLLRDA**S**TRDRHA-TQQEEVQLKHYTG-S---LK 822

O.latipes RSLQPGSETRPIRRHRGRSWKILF-----PEA**S**RRLQGS-------ESTEMSTLTV---- 779

**S.salar** TARLS-PSHSSRTLGKERSWRGFL-----GNV**S**RRQHTQPQHQIQVESTEMSSLSP--LS 802

O.mykiss TARLS-PTHSSRTLGRERSWRGFL-----GNV**S**RRQHTQPQQQTQVESTEMSSLSP--LN 803

D.rerio ------QDPSPANVQREPSR-GVL-----QTF**S**RRRRTQRAQTRE-----GHELSP--LA 807

C.auratus ------PVPSPTKLQRELSRRGLL-----QTF**S**-KRWTQRTQRRD-----VQELSP--LA 767

**.**

X.laevis EEDQEVTSKAE----- 838

G.gallus PEDCETLKESLAKSV- 843

H.sapiens PEDAEVFKSPAASGEK 839

R.norvegicus PEDAEVFKDSMVPGEK 838

O.latipes ---------------- 779

**S.salar** HV-------------- 804

O.mykiss HI-------------- 805

D.rerio EASSSV---------- 813

C.auratus EASSSV---------- 773

**Supplementary Figure 3. Alignment of Atlantic salmon *Salmo salar* TRPV4 amino acid sequences (XP_014016244.1) with the corresponding sequences from other vertebrates.**

The domains were determined using InterProScan. The blue lines mark the ankyrin repeat containing domains; the aa residues from the ankyrin repeats are in red. The dotted green lines correspond to the channel domains, with the corresponding transmembrane domains (S1 to S6 from top to the bottom) delimited by the red squares. Asterisk, fully conserved residues; column, residues displaying strongly similar properties; period, residues with weak similar properties. Clustal Omega (CLUSTAL O (1.2.4) multiple sequence alignment). *Oncorhynchus mykiss* (XP_036791455.1), *Danio rerio* (NP_001036195.1), *Carassius auratus* (XP_026117235.1), *Oryzias latipes* (XP_020561608.1), *Oreochromis mossambicus* (AGO02185.1), *Dicentrarchus labrax* (ADJ67990.1), *Xenopus tropicalis* (XP_002932129.1), *Gallus gallus* (NP_990023.1), *Homo sapiens* (NP_067638.3) and *Rattus norvegicus* (NP_076460.1).

In the rat, the LSRKFKD sequence is a putative arachidonate binding site, while the glutamate residue at the C-terminal end is important for the channel inactivation (E-827); the highly conserved aspartate and methionine residues, located in the pore region (from L-701 to L-713) are determinants of Ca^2+^ permeability (Voets et al., 2002; Nilius et al., 2003; Voets and Nilius, 2003; White et al., 2016). The aspartate **D**-682 is important for the affinity of TRPV4 channel to the blocker ruthenium red, while the **Y**555 is essential for the response to heat and to the agonist 4αPDD (Vriens et al., 2004). As shown in the human TRPV4 the conserved amino acid residues in the transmembrane domain 5 are also important determinants of the response to 4αPDD (F617, Y621, F624) as well as to hypotonic stimulation (Y621, F624) and temperature (Y617) (Klausen et al., 2014). The conserved aromatic residue Y702, in transmembrane domain 6 is involved in channel activation (Klausen et al., 2014).

**Supplementary Figure 3. TRPV4 Protein Alignment**

D.rerio ------------------MTESLSVSSPPDNSAQDS---------------SEAADGDPN 27

C.auratus MTEQGFSASTLLKRYYLAMTESNSISSPSGAAAEDLSDGK-----------DLTADGDPN 49

G.gallus ------------------------------------------MADPEDPRDAGDVLGDDS 18

H.sapiens ------------------MA------------DSSEGPRAGPGEVAELPGDESGTPGGEA 30

R.norvegicus ------------------MA------------DPGDGPRAAPGDVAEPPGDESGTSGGEA 30

X.tropicalis ------------------MA--DPSHLLKHNASVDIDDSQGD----------DGSNHNDS 30

**S.salar** -MNEDRSPATLLRRCRIAMTETDTLHSDANKAALSSGSGEGGGS-----GEGQPDADGTC 54

O.mykiss -MNENRSPATLLRRCRIAMTETDTPPSAANKAALSSGSGEGGGS-----GEGQPDADGTC 54

O.latipes -MNEGR--SALLRRCHLAFSRADTLGSAGSAASEDCKEG----------DAAQAEGEAA- 46

O.mossambicus -MNEGR--SAIFKRRHLALPKGNAISSEPS-ISVDLGDS----------EAAQPEGDGA- 45

D.labrax ------------------------------------------------------------ 0

D.rerio FPMSSMAALLENDDVSQPTHE--------LPRPGQQNDQKQNMRIRFPGPFKKGVPNPMD 79

C.auratus FPMSSLAELLDNDDGSQPPQD--------SARPGLQNDNKQNSRIRFPGAFKKGVPNPMD 101

G.gallus FPLSSLANLFEVEDTPSPAEP--------SRGPPGAGDGKQNLRMKFHGAFRKGPPKPME 70

H.sapiens FPLSSLANLFEGEDGSLSPSPA------DASRPAGPGDGRPNLRMKFQGAFRKGVPNPID 84

R.norvegicus FPLSSLANLFEGEEGSSSLSPV------DASRPAGPGDGRPNLRMKFQGAFRKGVPNPID 84

X.tropicalis FPLSSLANLFENEESSAPNE--------GVRSPQVPGDNKQNLRIRFQGPFRKGISNPMD 82

**S.salar** PDLSALADLFESEEGSQSPQDPAPDV--DRPGQLQPGDSRQNLRMKFHGAFKKGISNPMD 112

O.mykiss PDLSALADLFESEEGSQSPQDPAPDI--DRPGQLQPGDGRQNLRMKFHGAFKKGISNPMD 112

O.latipes FPLSELSQLFESEDGSQSAQDTSQESALELVQPGNPADSRQNLRTKFQGAFRKGISHPMD 106

O.mossambicus FPLSEFSHLFESQDGSPATQDSSQESILEPAQPGHPADSRQYLRMKFHGAFKKGISNPMD 105

D.labrax ------------------------------------------------------------ 0

D.rerio LLESDYTEYP-----KQAPMDSMFDYGTCRQINNNKKGRRKKLPRGKAEIGMSCDE---- 130

C.auratus LLESTMSEYPVAPGPKKAPMDSLFDYGTCRELNNHK-KRRKKLPRGKAEIEMSCDE---- 156

G.gallus LLESTIYESSVVPAPKKAPMDSLFDYGTYRQHPSEN-KRWRRRVVEKPV------AGTKG 123

H.sapiens LLESTLYESSVVPGPKKAPMDSLFDYGTYRHHSSDN-KRWRKKIIEKQP------QSPKA 137

R.norvegicus LLESTLYESSVVPGPKKAPMDSLFDYGTYRHHPSDN-KRWRRKVVEKQP------QSPKA 137

X.tropicalis LLESTIYESS---APKKAPMDSLFGYETYHHHPTEN-RRKRKKILLEK-----ENLNSQA 133

**S.salar** LLESTIYESPVAPGPKKAPMDSLFDYGTYRHTNNKK-PRRKKLPRGKTET--SCNESLDP 169

O.mykiss MLESTIYESPVAPGPKKAPMDSLFDYGTYRHTNNKK-PRRKKLPRGKTET--SCNESLDP 169

O.latipes LFEATIYESNVVPAPKKAPMDSLFDYGTYGNSSNQK-KRRKKLPRGKTEA--SCDIV--- 160

O.mossambicus LLESTIYESNVVPAPKKAPMDSLFDYGTYGNSSNQK-KRRKKLPKGKTEA--SCDES--- 159

D.labrax ------------------------------------------------------------ 0

D.rerio -GSPEPPVLKVFNRWMLFEAVSRADPRALDGLLQYLQSHEKRLTDEEFKELSTGKTCLPK 189

C.auratus -GSPEPPVLKVFNRWLLFEAVSRADRRALDGLLQYLQSHEKRLIDEEFKEPSTGKTCLPK 215

G.gallus PAPNPPPVLKVFNRPILFDIVSRGSPDGLEGLLSFLLTHKKRLTDEEFREPSTGKTCLPK 183

H.sapiens PAPQPPPILKVFNRPILFDIVSRGSTADLDGLLPFLLTHKKRLTDEEFREPSTGKTCLPK 197

R.norvegicus PAPQPPPILKVFNRPILFDIVSRGSTADLDGLLSYLLTHKKRLTDEEFREPSTGKTCLPK 197

X.tropicalis PSPDPPPVIKMFNRHMLFDIVSRGSTAELEGFLPFLLAQKKRLTDEEFREASTGKTCLTK 193

**S.salar** PGLDPPKVLKVFNRMLLFDGVSRADPEALSGLLEYLQGHEKRLTDEEFKEPSTGKTCLPK 229

O.mykiss PGLDPPKVLKVFNRVLLFDGVSRADPEALSGLLEYLQGHEKRLTDEEFKEPSTGKTCLPK 229

O.latipes PNPDPPKVMKIFNRILLFDCVSRGDPEDLEGLLEYLQVHEKRLTDEEFREPSTGKTCLPK 220

O.mossambicus QSSDPPKVVKVFNRSLLFDCVSRGDPGELEGLLEYLQSNNKRLTDEEFREPYTGKTCLPK 219

D.labrax ------------------------------------------------------------ 0

D.rerio ALLNLHNGQNDTIPILVDIAEQTGNLREFINTPFRDVYYRGQMALHIAIERRCKQYVELL 249

C.auratus ALLNLHNGHNDTIPVLVDIAEKTGNLREFINTPFRDVYYRGQTALHIAIERRCKQYVELL 275

G.gallus ALLNLSAGRNDTIPILLDIAEKTGNMREFINSPFRDVYYRGQTALHIAIERRCKHYVELL 243

H.sapiens ALLNLSNGRNDTIPVLLDIAERTGNMREFINSPFRDIYYRGQTALHIAIERRCKHYVELL 257

R.norvegicus ALLNLSNGRNDTIPVLLDIAERTGNMREFINSPFRDIYYRGQTALHIAIERRCKHYVELL 257

X.tropicalis ALMNLNGGKNDTIPMLIDIAEKTGNLREFINSPFRDVYYRGQTALHIAIERRCKHYVELL 253

**S.salar** ALLNLYSGQNDTIPMLMDIAEQTVNLHEFINTPFRDVYYRGQTALHIAIERRCKQYVELL 289

O.mykiss ALLNLYSGQNDTIPMLMDIAEQTVNLHEFINTPFRDVYYRGQTALHIAIERRCKQYVELL 289

O.latipes ALLNLYGGRNNTIPLLVDIAEKTGNLREFINTPFRDVYYRGQTALHIAIERRCKHYVELL 280

O.mossambicus ALMNLYGRQNNTIPVLVDIAEKNGSLREFINTPFRDVYYRGQTALHIAIERRCKQYVKLL 279

D.labrax ------------------------------------------------------------ 0

D.rerio VEKGADVHAQARGRFFQPRDEGGYFYFGELPLSLAACTNQPDMVHYLTENGHKKADLRRQ 309

C.auratus VEKGADVHAQARGRFFQPREEGGYFYFGELPLSLAACTNQPDMVHYLTENSHKMADLRRQ 335

G.gallus VEKGADVHAQARGRFFQPKDEGGYFYFGELPLSLAACTNQPHIVHYLTENGHKQADLRRQ 303

H.sapiens VAQGADVHAQARGRFFQPKDEGGYFYFGELPLSLAACTNQPHIVNYLTENPHKKADMRRQ 317

R.norvegicus VAQGADVHAQARGRFFQPKDEGGYFYFGELPLSLAACTNQPHIVNYLTENPHKKADMRRQ 317

X.tropicalis VEKGADVHAQARGRFFQPKDEGGYFYFGELPLSLAACTNQPDIVHYLTENAHKKADIRRQ 313

**S.salar** VEKGADVHAQARGRFFQPRDEGGYFYFGELPLSLAACTNQPNMVHYLTENAHKKADLRRQ 349

O.mykiss VEKGADVHAQARGRFFQPRDEGGYFYFGELPLSLAACTNQPNMVHYLTENAHKKADLRRQ 349

O.latipes MEKGADVHAQARGRFFEPKDEGGYFYFGELPLSLAACTNQPNIVNYLTENPHKKADLRRQ 340

O.mossambicus VEKGADVHAQARGRFFQPKDEGGYFYFGELPLSLAACTNQPDIVHYLTENPHKKADVRRQ 339

D.labrax -----------------------YFYFGELPLSLAACTNQPDIVHYLTENPHKKADLRRQ 37

******************.:*:***** ** **:***

D.rerio DSRGNTVLHALVHIADNTRDNTRFVTKMFDLLLIKCAKLYPDCNLENILNNDGMSPLMMA 369

C.auratus DSRGNTVLHALVHIADNTRDNTRFVTKMYDLLLIKCAKLYPDCNFENILNNDGMSPLMMA 395

G.gallus DSRGNTVLHALVAIADNTRENTKFVTKMYDLLLIKCAKLFPDTNLEALLNNDGLSPLMMA 363

H.sapiens DSRGNTVLHALVAIADNTRENTKFVTKMYDLLLLKCARLFPDSNLEAVLNNDGLSPLMMA 377

R.norvegicus DSRGNTVLHALVAIADNTRENTKFVTKMYDLLLLKCSRLFPDSNLETVLNNDGLSPLMMA 377

X.tropicalis DSRGNTVLHALVAIADNTRENTKFVTKVYDLLVIKCVKLYPDSSLEAIFNNDSMSPLMMA 373

**S.salar** DSRGNTVLHALVHIADNTRDNTRFLTKMYDLLLTKCAKLYPECSLEDILNNDGMSPLMMA 409

O.mykiss DSRGNTVLHGLVHIADNTRDNTRFLTKMYDLLLTKCAKLYPECSLEDILNNDGMSPLMMA 409

O.latipes DSRGNTALHALVHIADNTKDNTRFLTKMYDLLLIKCTKLYPECNLEKMANNDGLTPLMMA 400

O.mossambicus DSRGNTVLHALVHIADNTKDNTRFLTKMYDLLLIKTAKLYPDCNLETVPNNDGMSPLMMA 399

D.labrax DSRGNTVLHALVHIADNTKDNTRFLTKMYDLLLIKSAKLYPDCSLETVLNNDGMSPLMMA 97

******.**.** *****::**:*:**::***: * :*:*: .:* : ***.::*****

D.rerio AKLGKIGVFQHTIRREIKDEEARH**LSRKFKD**WAYGPVYSNLYDLSSLDTCGEEVSVLEIL 429

C.auratus AKLGKIGVFQHIIRREIKDEEARH**LSRKFRD**WAYGPVYSNLYDLSSLDTCGEEVSVLEIL 455

G.gallus AKTGKIGIFQHIIRREIADEDVRH**LSRKFKD**WAYGPVYSSLYDLSSLDTCGEEVSVLEIL 423

H.sapiens AKTGKIGIFQHIIRREVTDEDTRH**LSRKFKD**WAYGPVYSSLYDLSSLDTCGEEASVLEIL 437

R.norvegicus AKTGKIGVFQHIIRREVTDEDTRH**LSRKFKD**WAYGPVYSSLYDLSSLDTCGEEVSVLEIL 437

X.tropicalis AKLGKIGIFQHIIRLEIKDEEARH**LSRKFRD**WAYGPVYSSLYDLSMLDTCGEEVSVLEIL 433

**S.salar** AKLGKIGVFQHIIRREIKDEEARH**LSRKFKD**WAYGPVYSSLYDLSSLDTCGEEVSVLEIL 469

O.mykiss AKLGKIGVFQHIIRREIKDEEARH**LSRKFKD**WAYGPVYSSLYDLSSLDTCGEEVSVLEIL 469

O.latipes AKLGKIGVFQHIIRREIKDEEVRH**LSRKFKD**WAYGPVYSSLYDLSSLETCGEEPSVLEIL 460

O.mossambicus ARLGKIGIFQHIIRREIKDEEVRH**LSRKFKD**WAYGPVYSSLYDLSSLDTCGKESSVLEIL 459

D.labrax AKLGKIGVFQHIIRREVKDEEVRH**LSRKFKD**WAYGPVYSSLYDLSSLDTCGEEPSVLEIL 157

*: ****:*** ** *: **:.*******:*********.***** *:***:* ******

D.rerio VYNSKIENRHEMLAVEPINELLRAKWQKFAAVTFYISVFSYLVTMIIFTLVAYYRPSVGK 489

C.auratus VYNSKIENRHEMLAVEPINELLRAKWQKFAAVTFYISVFSYLVTMIIFTLVAYYRPSVGT 515

G.gallus VYNSKIENRHEMLAVEPINELLRDKWRKFGAVSFYISVVSYLCAMIIFTLIAYYRPMEGP 483

H.sapiens VYNSKIENRHEMLAVEPINELLRDKWRKFGAVSFYINVVSYLCAMVIFTLTAYYQPLEGT 497

R.norvegicus VYNSKIENRHEMLAVEPINELLRDKWRKFGAVSFYINVVSYLCAMVIFTLTAYYQPLEGT 497

X.tropicalis VYNSKVENRHEMLAVEPINELLRDKWQKFGAVSFYISVVSYLIAMIIFTLIAYYRPMDGT 493

**S.salar** VYNSRIENRHEMLAVEPINELLRVKWQKFAAVTFYISVVSYLVTMIIFTLVAYYRPSQGM 529

O.mykiss VYNSRIENRHEMLAVEPINELLRAKWQKFAAVTFYISVVSYLVTMIIFTLVAYYRPSQGT 529

O.latipes VYNSRNENCHEMLAVEPINELLRAKWQKFAAVTFYISVVSYLITMIIFTLVAYYHPTEGK 520

O.mossambicus VYTSHNENRHEMLAVEPINELLRAKWNRFAAVTFYISVFSYLITMIIFTLVAYYQPTDGK 519

D.labrax VYNSRNENRHEMLAVEPINELLRAKWQKFAAVTFYISVVSYLITMIIFTLVAYYHPTQGK 217

**.*: ** ************** **.:*.**:***.*.*** :*:**** ***:* *

D.rerio PPYAYDTTEDKVRLGGEIITVGSGLFFFVTNIKDLFLKKCPGVNSIFVDGSFQLLYFI**Y**S 549

C.auratus PPYDYSTTEAKVRLAGEIITVASGVFFFVTNIKDLFLKKCPGVNSLFIDGSFQFLYFI**Y**S 575

G.gallus PPYPYTTTIDYLRLAGEIITLLTGILFFFSNIKDLFMKKCPGVNSFFIDGSFQLLYFI**Y**S 543

H.sapiens PPYPYRTTVDYLRLAGEVITLFTGVLFFFTNIKDLFMKKCPGVNSLFIDGSFQLLYFI**Y**S 557

R.norvegicus PPYPYRTTVDYLRLAGEVITLLTGVLFFFTSIKDLFMKKCPGVNSLFVDGSFQLLYFI**Y**S 557

X.tropicalis PPYPYRTTMDYMRLAGEIVTLLTGVVFFITNIKDLFMKKCPGVNSLFIDGSFQLLYFI**Y**S 553

**S.salar** PPYPYTTSTDYLRLGGEVITLGSGVFFFLTNIKDLFLKKCPGVNSLFVDGSFQLLYFI**Y**S 589

O.mykiss PPYPYTTSTDYLRLGGEVITLGSGVFFFLTNIKDLFLKKCPGVNSLFVDGSFQLLYFI**Y**S 589

O.latipes PPFPYTTSTDYLRMVGEIFTLASGIFFFLTNIKDLFLKKRPGVKSLVMDGSFQLLYFI**Y**S 580

O.mossambicus PPYPHTTSSDYWRMAGEIVTLASGIFFFLTNIKDLFLKKCPGVKSLFIDGSFQLLYFI**Y**S 579

D.labrax PPYPYTTSSDYLRMAGEIVTLTSGIFFFLTNIKELFLKKCQGVKSLFIDGSFQLLYFI**Y**S 277

**: : *: *: **:.*: :*:.**.:.**:**:** **:*:.:*****:******

D.rerio VLVVGSAALYLSGIEAYVSVMVFALTLGGMNPLYFTRGLKLTGTYSIMIQKILIKDLFRF 609

C.auratus VLVLVSAALYLSGIEAYVSVMVFALALGWMNTLYFTRGLKLTGTYSIMIQKILIKDLFRF 635

G.gallus VLVIVTAGLYLGGVEAYLAVMVFALVLGWMNALYFTRGLKLTGTYSIMIQKILFKDLFRF 603

H.sapiens VLVIVSAALYLAGIEAYLAVMVFALVLGWMNALYFTRGLKLTGTYSIMIQKILFKDLFRF 617

R.norvegicus VLVVVSAALYLAGIEAYLAVMVFALVLGWMNALYFTRGLKLTGTYSIMIQKILFKDLFRF 617

X.tropicalis VLVIITAVLYLVGIESYLAVMVFALVLGWMNALYFTRGLKLTGTYSIMLQKILFKDLFRF 613

**S.salar** VLVIVTAALYLSGIEAYVSVMVFALVLGWMNTLYFTRGLKLTGTYSIMIQKILFKDLFRF 649

O.mykiss LLVIVTAALYLSGIEAYLSVMVFALVLGWMNTLYFTRGLKLTGTYSIMIQKILFKDLFRF 649

O.latipes ILIIITAALYLSGIKAYVSVMVFALVLGWMNTLYFTRGLKLTGTYSIMIQKILLKDIFRF 640

O.mossambicus VLIIVTAALYLSGIEAYVSVMVFALALGWMNTLYFTRGLKLTGTYSIMIQKILFKDLFRF 639

D.labrax VLIVVTAALYLSGIEAYVSVMVFALVLGWMNTLYFTRGLKLTGTYSIMIQKILFKDLFRF 337

:*:: :* *** *:::*::******.** ** ****************:****:**:***

D.rerio LLVYVLFMIGYASALVSLLTICPNKDTCK-----ENCPTYPECRDTNTFSEFLL**D**LFKLT 664

C.auratus LLVYVLFMIGYASALVSLLTICPDQKTCK-----DSCPKYPECRDTNTFSEFLL**D**LFKLT 690

G.gallus LLVYLLFMIGYASALVSLLNPCPSSESCSEDHSNCTLPTYPSCRDSQTFSTFLL**D**LFKLT 663

H.sapiens LLVYLLFMIGYASALVSLLNPCANMKVCNEDQTNCTVPTYPSCRDSETFSTFLL**D**LFKLT 677

R.norvegicus LLVYLLFMIGYASALVTLLNPCTNMKVCNEDQSNCTVPSYPACRDSETFSAFLL**D**LFKLT 677

X.tropicalis LLVYLLFMIGYASALVSLLNPCTSQESCIETSSNCTVPEYPSCRDSSTFSKFLL**D**LFKLT 673

**S.salar** LLVYVLFMIGYSSALVSLLAVCPGPDEVCPEE--GGCPTYPQCRDTDTFSNFLL**D**LFKLT 707

O.mykiss LLVYVLFMIGYSSALVSLLAVCPGPDEVCPEE--GGCPTYPQCRDTDTFSNFLL**D**LFKLT 707

O.latipes LLVYLLFMIGYASALVSLLTVCPTSGPEC--E--GGCPTYPKCREPGTFSTFLL**D**LFKLT 696

O.mossambicus LLVYVLFMIGFASALVSLLTVCPPPGTVC--N--GSCPTYPACRDNNTFSAFLL**D**LFKLT 695

D.labrax LLVYVLFMIGYASALVSLLTVCPPLGTEC--D--GGCPTHPNCRDPDTFSTFLL**D**LFKLT 393

****:*****::****:** * * :* **: *** *********

D.rerio IGIG**D**LDNMLKGAQYPAVFLILLVTYIILTFVPLLNMLIALMGETVGQVSKESKKIWKLQ 724

C.auratus IGIGELDDMLKGAQYPVVFLILLVTYIILTFVLLLNMLIALMGETVGQVSKESKQIWKLQ 750

G.gallus IG**M**G**D**LE-MLESAKYPGVFIILLVTYIILTFVLLLNMLIALMGETVGQVSKESKHIWKLQ 722

H.sapiens IG**M**G**D**LE-MLSSTKYPVVFIILLVTYIILTFVLLLNMLIALMGETVGQVSKESKHIWKLQ 736

R.norvegicus IG**M**G**D**LE-MLSSAKYPVVFILLLVTYIILTFVLLLNMLIALMGETVGQVSKESKHIWKLQ 736

X.tropicalis IG**M**G**D**LE-MINSAKYPAVFIILLVTYIILTFVLLLNMLIALMGETVGQVSKESKQIWKLQ 732

**S.salar** IG**M**G**D**LD-MVSSAQYPAVFLILLVTYIILTFVLLLNMLIALMGETVSQVSKESKKIWKLQ 766

O.mykiss IG**M**G**D**LD-MVSSAQYPAVFLILLVTYIILTFVLLLNMLIALMGETVSQVSKESKKIWKLQ 766

O.latipes IG**M**G**D**LE-MINSAQYPEVFLILLVTYIILTFVLLLNMLIALMGETVGQVSKESKKIWKLQ 755

O.mossambicus IG**M**G**D**LD-MIYSAQNPVVFLILLVTYIILTFVLLLNMLIALMGETVGQVSKESKKIWKLQ 754

D.labrax IG**M**GELD-MIHSAKYPAVFLILLVTYIILTFVLLLNMLIALMGETVGQVSKESKKIWKLQ 452

**:*:*: *: .:: * **::*********** *************.*******:*****

D.rerio WATTILDIERSFPVCLRRSFRVGEMVTVGKGLDGKPDKRWCFRVDEVKWSHWNQNLGIIN 784

C.auratus WATTILDIERSFPVCLRKSFRVGEMVTVGKGLDGTPDKRWCFRVDEVKWSHWNQNLGIIN 810

G.gallus WATTILDIERSFPLFLRRAFRSGEMVTVGKGTDGTPDRRWCFRVDEVNWSHWNQNLGIIS 782

H.sapiens WATTILDIERSFPVFLRKAFRSGEMVTVGKSSDGTPDRRWCFRVDEVNWSHWNQNLGIIN 796

R.norvegicus WATTILDIERSFPVFLRKAFRSGEMVTVGKSSDGTPDRRWCFRVDEVNWSHWNQNLGIIN 796

X.tropicalis WATTILDIERSFPVCMRKAFRSGEMVTVGKNLDGTPDRRWCFRVDEVNWSHWNQNLGIIN 792

**S.salar** WATTILDIERSFPVCLRKSFRSGEMVTVGKNWDGTPDRRWCFRVDEVNWCHWNQNLAIIN 826

O.mykiss WATTILDIERSFPVCLRKSFRSGEMVTVGKNWDGTPDRRWCFRVDEVNWCHWNQNLAIIN 826

O.latipes WATTILDIEHSFPVCLRRSFRVGEMVTVGKNLDGTPDRRWCFRVDEVNWCHWNQNLAIIN 815

O.mossambicus WATTILDIERSFPVCLRKSFRVGEMVTVGKNYDGTPDRRWCFRVDEVNWCHWNQNLAIIN 814

D.labrax WATTILDIERSFPVCLRKSFRAGEMVTVGKNWDGTPDRRWCFRV---------------- 496

*********:***: :*::** ********. **.**:******

D.rerio **E**DPGQKDLSE------HTQGGRGLRRDRWSTVVPRVVELNRGSRDH--TVEMEPLTGRHR 836

C.auratus **E**DPGQKDHYE------QTQGGRGLRRDRWSTVVPRVVELNRGSRDH--ILEMEPLTGRHR 862

G.gallus **E**DPGKSDTYQ---YYGFSHTVGRLRRDRWSTVVPRVVELNKSCPTEDVVVPLGTMGT-AE 838

H.sapiens **E**DPGKNETYQ---YYGFSHTVGRLRRDRWSSVVPRVVELNKNSNPDEVVVPLDSMGN-PR 852

R.norvegicus **E**DPGKSEIYQ---YYGFSHTMGRLRRDRWSSVVPRVVELNKNSGTDEVVVPLDNLGN-PN 852

X.tropicalis **E**DPGRNDGYQ---YYGFSQTVGRLRRDRWSVVVPRVVELNKAPQHSDDVVVPLGNIPQVQ 849

**S.salar** **E**DPGKNITETQQCSGTVHQTVRGLRRDRWSTVVPRVVEQNKGPRPRDLVLEMEPLTPRHR 886

O.mykiss **E**DPGKNITETQQCPGTVHQTVRGLRRDRWSTVVPRVVEQNKGPRPRDLVLEMEPLTPRHR 886

O.latipes **E**DPGRSDTSQ---TNGLRQSVKGLRRDRWTTVVPRVMELSKSPQPHDLVVEMEPLTTRN- 871

O.mossambicus **E**DPGKSETIQ---ANGLQQGVRALRRDRWSTVVPRAVELSKGSQSHDLAVEMEPLSPRH- 870

D.labrax ------------------------------------------------------------ 496

D.rerio LKSES-------------- 841

C.auratus HKSES-------------- 867

G.gallus ARERRHGQTP-----SSPL 852

H.sapiens CDGHQQGYPRKWRTDDAPL 871

R.norvegicus CDGHQQGYAPKWRAEDAPL 871

X.tropicalis TYSQRQENAQNWKKDETHI 868

**S.salar** PCAEG-------------- 891

O.mykiss PFAEG-------------- 891

O.latipes ------------------- 871

O.mossambicus ------------------- 870

D.labrax ------------------- 496

**Supplementary Figure 4. Variations of TRPV1 and TRPV4 mRNA relative abundance in different tissues of *S. salar*.**

Messenger RNA of TRPV channels were identified in February and July in all tissues investigated. From left to right: **P**, pineal organ; **R**, retina, **Te**, telencephalon; **Di**, diencephalon; **OT**, *optic tectum*; **Ce**, cerebellum; **Pit**, pituitary; **H**, heart; **Sk**, skin (including the lateral line); **AdF**, adipose fin; **Li**, liver; **Int**, intestine; **Gi**, gills; **Ki**, kidney; **Spl**, spleen; **Blo**, blood. Data are replotted from Figure 1.

**Supplementary References**

Cordero-Morales, J.F., and Vasquez, V. (2018). How lipids contribute to ion channel function, a fat perspective on direct and indirect interactions. *Current Op. Struct. Biol.* 51**,** 92-98.

Cohen, M.R., and Moiseenkova-Bell, V.Y. (2014). "Structure of Thermally Activated TRP Channels," in *Thermal Sensors,* eds. L.D. Islas & F. Qin.), 181-211.

Klausen, T.K., Janssens, A., Prenen, J., Owsianik, G., Hoffmann, E.K., Pedersen, S.F., and Nilius, B. (2014). Single point mutations of aromatic residues in transmembrane helices 5 and-6 differentially affect TRPV4 activation by 4 alpha-PDD and hypotonicity: Implications for the role of the pore region in regulating TRPV4 activity. *Cell Calcium* 55, 38-47.

Leonelli, M., Graciano, M.F.R., and Britto, L.R.G. (2011). TRP channels, omega-3 fatty acids, and oxidative stress in neurodegeneration: from the cell membrane to intracellular cross-links. *Brazilian J. Med. Biol. Res.* 44**,** 1088-1096.

Nilius, B., Watanabe, H., and Vriens, J. (2003). The TRPV4 channel: structure-function relationship and promiscuous gating behaviour. *Pflugers Arch.* 446**,** 298-303.

Shuba, Y.M. (2020). Beyond Neuronal Heat Sensing: Diversity of TRPV1 Heat-Capsaicin Receptor-Channel Functions. *Front. Cell. Neurosci.* 14**,** 612480.

Voets, T., and Nilius, B. (2003). The pore of TRP channels: trivial or neglected? *Cell Calcium* 33**,** 299-302.

Voets, T., Prenen, J., Vriens, J., Watanabe, H., Janssens, A., Wissenbach, U., Bodding, M., Droogmans, G., and Nilius, B. (2002). Molecular determinants of permeation through the cation channel TRPV4. *J. Biol. Chem.* 277**,** 33704-33710.

Vriens, J., Watanabe, H., Janssens, A., Droogmans, G., Voets, T., and Nilius, B. (2004). Cell swelling, heat, and chemical agonists use distinct pathways for the activation of the cation channel TRPV4. *PNAS* 101, 396-401.

White, J.P., Cibelli, M., Urban, L., Nilius, B., Mcgeown, J.G., and Nagy, I. (2016). TRPV4: Molecular conductor of a diverse orchestra. *Physiol. Rev.* 96**,** 911-973.
