# Supplementary figures and images for: Transient Receptor Potential-Vanilloid (TRPV1-TRPV4) Channels in the Atlantic Salmon, Salmo salar. A Focus on the Pineal Gland and Melatonin Production
Source: Front Physiol. 2022 Jan 7;12:784416. doi: 10.3389/fphys.2021.784416 (PMC8782258; doi:10.3389/fphys.2021.784416)

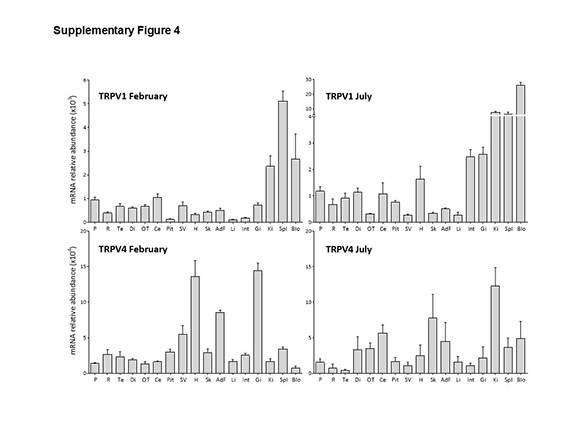

Supplement: Supplementary file 2 [file Image_1.JPEG]
